# Supplementary material for: Urea-formaldehyde resin room temperature phosphorescent material with ultra-long afterglow and adjustable phosphorescence performance
Source: Nat Commun. 2024 May 24;15:4415. doi: 10.1038/s41467-024-48744-w (PMC11126683; doi:10.1038/s41467-024-48744-w)
Supplement: Supplementary file 1 — Supplementary Information [file 41467_2024_48744_MOESM1_ESM.pdf]

Supplementary Information

Urea-formaldehyde resin room temperature phosphorescent material with ultra-long afterglow and adjustable phosphorescence performance

Wensheng Xu[1], Bowei Wang *[1,2,3,4], Shuai Liu[5], Wangwang Fang[1,2,5], Qinglong Jia[1], Jiayi Liu[1], Changchang Bo[1], Xilong Yan[1,2,3,4], Yang Li[1,3], and Ligong Chen *[1,2,3,4]

1 School of Chemical Engineering and Technology, Tianjin University, Tianjin, 300350, People's Republic of China.
2 Zhejiang Institute of Tianjin University, Shaoxing, 312300, P.R. China.
3 Collaborative Innovation Center of Chemical Science and Engineering (Tianjin), Tianjin 300072, P. R. China.
4 Tianjin Engineering Research Center of Functional Fine Chemicals, Tianjin 300350, P.R. China.
5 Shaoxing Xingxin New Materials Co., Ltd, Shaoxing, Zhejiang, P. R. China.

* Corresponding author at: School of Chemical Engineering and Technology, Tianjin University, Tianjin 300350, P. R. China.

E-mail: bwwang@tju.edu.cn (Bowei Wang, ORCID ID: 0000-0002-9400-0698); lgchen@tju.edu.cn (Ligong Chen, ORCID ID: 0000-0002-3442-5694).

Contents

1. Supplementary Methods 1
2. Supplementary Discussion 8
2.1 Phosphorescent emission/decay mechanism and excellent polymer matrix. 8
2.2 The measurement and characterization of UF-RTPs. 9
2.3 The measurement and characterization of diaminobenzene derivatives in UF-RTPs. 14
2.4 Analysis of ESP distribution and poles of UF chains and the guest molecules. 18
2.5 Quantum chemical calculations of guest molecules. 22
2.6 The photophysical property of UF-RTPs. 23
2.7 Thes luminescence mechanism of aromatic *o*-diamine in UF-RTPs. 27
2.8 Stability of UF-RTPs. 31
2.9 The measurement and characterization of μUFs. 33
Supplementary References 39

1. Supplementary Methods

Monitoring the UF-RTPs formation process by IR

Monitoring the copolymerization will help to understand the formation mechanism of UF-RTPs. However, the high reaction temperature causes the reaction rate to be too fast to monitor. Therefore, we monitored the formation process of UF-RTPs by reducing the reaction temperature and extending the reaction time.

2.0 g urea and 2.0 mL deionized water were added to a 100 mL one necked flask and heated to 100°C. Then 15.0 mg 14DAP was mixed with 1.0 g paraformaldehyde powder and added to the above reaction mixture. The resulting mixture was continuously stirred with a glass rod for 15 min and sampled every 5 minutes for analysis.

Quantum chemical calculation

Gaussain 09 was used to optimize the configuration of guest molecules and their derivatives under the b3lyp/6-31g(d). The singlet, triplet, binding energy and SOC of guest molecules and matrix were also calculated under b3lyp/def2-tzvp by ORCA 5.0[1]. The ESP distribution and poles of the molecules were calculated using Multifunctional Wavefunction Analyzer (Mutiwfn)[2].

Synthesis

1H-phenanthro[9,10-d]imidazole: 9,10-Diaminophenanthine (100.0 mg, 0.48 mmol), 40% formaldehyde aqueous solution (100 μL, 1.45 mmol) and methanol (20.0 mL) were added to a one-neck flask of 50 mL. The mixture was stirred at 60°C for 4 h. Then, the solid was collected by filtration and washed with water, dried at 50°C for 1 h to obtain 87.1 mg gray solids with a yield of 83%. ^{1}H NMR (600 MHz, DMSO-d6): δ 7.94 (s, 2H), 7.55 (s, 2H), 7.40 (s, 1H), 6.81 (s, 2H), 6.72 (s, 2H). ^{13}C NMR (151

MHz, DMSO-d6) δ 139.77, 127.9, 127.58, 125.58, 124.40, 122.23. ESI-MS, m/z: $[M+1]^+$, calcd. for $C_{15}H_{11}N_2$, 219.1. Found, 219.2.

1H-naphtho[2,3-d]imidazole: 2, 3-Diaminonaphthalene (500.0 mg, 3.16 mmol) and formic acid (10 mL) were added to a 50 mL round-bottom flask equipped with a reflux condensing tube. The mixture was heated to reflux for 6 h, and the reaction was monitored by TLC. After completion of the reaction, it was cooled to room temperature and poured into a 100 mL saturated Na_2CO_3 aqueous solution. The resulting mixture was stirred for 10 min and then filtered to collect the precipitated solids. The solids were washed with water and dried under a vacuum to obtain 414.1 mg desired product with a yield of 78%.^{1}H NMR (600 MHz, Chloroform-d): δ 8.36 (s, 1H), 8.10 (s, 2H), 7.93 (s, 2H), 7.42 (s, 2H). ^{13}C NMR (151 MHz, Chloroform-d): δ 144.34, 130.83, 128.05, 124.62, 111.93. ESI-MS, m/z: $[M+1]^+$, calcd. for $C_{11}H_9N_2$, 169.1. Found, 169.1.

Supplementary Figures

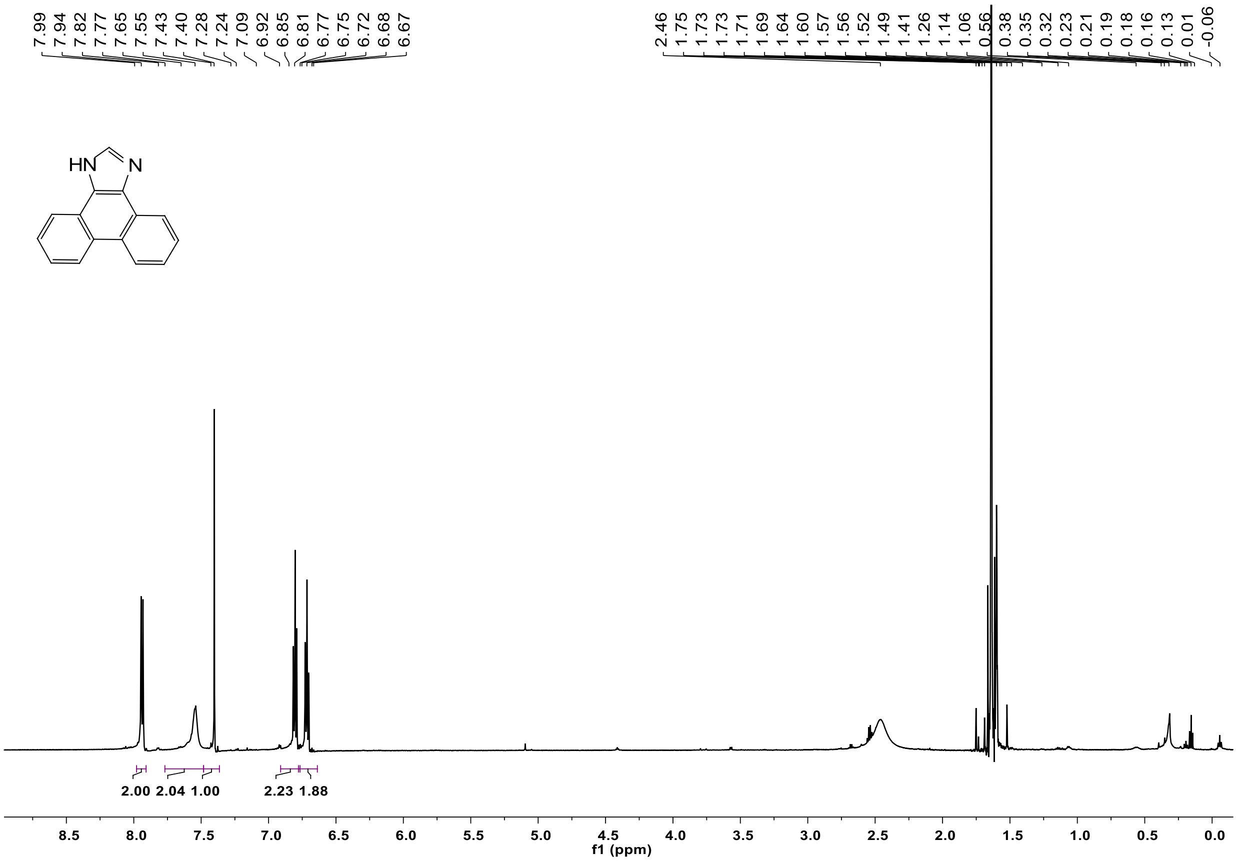

Supplementary Figure 1. ^{1}H NMR spectrum (600 MHz, DMSO-d6) of 1H-phenanthro[9,10-d]imidazole.

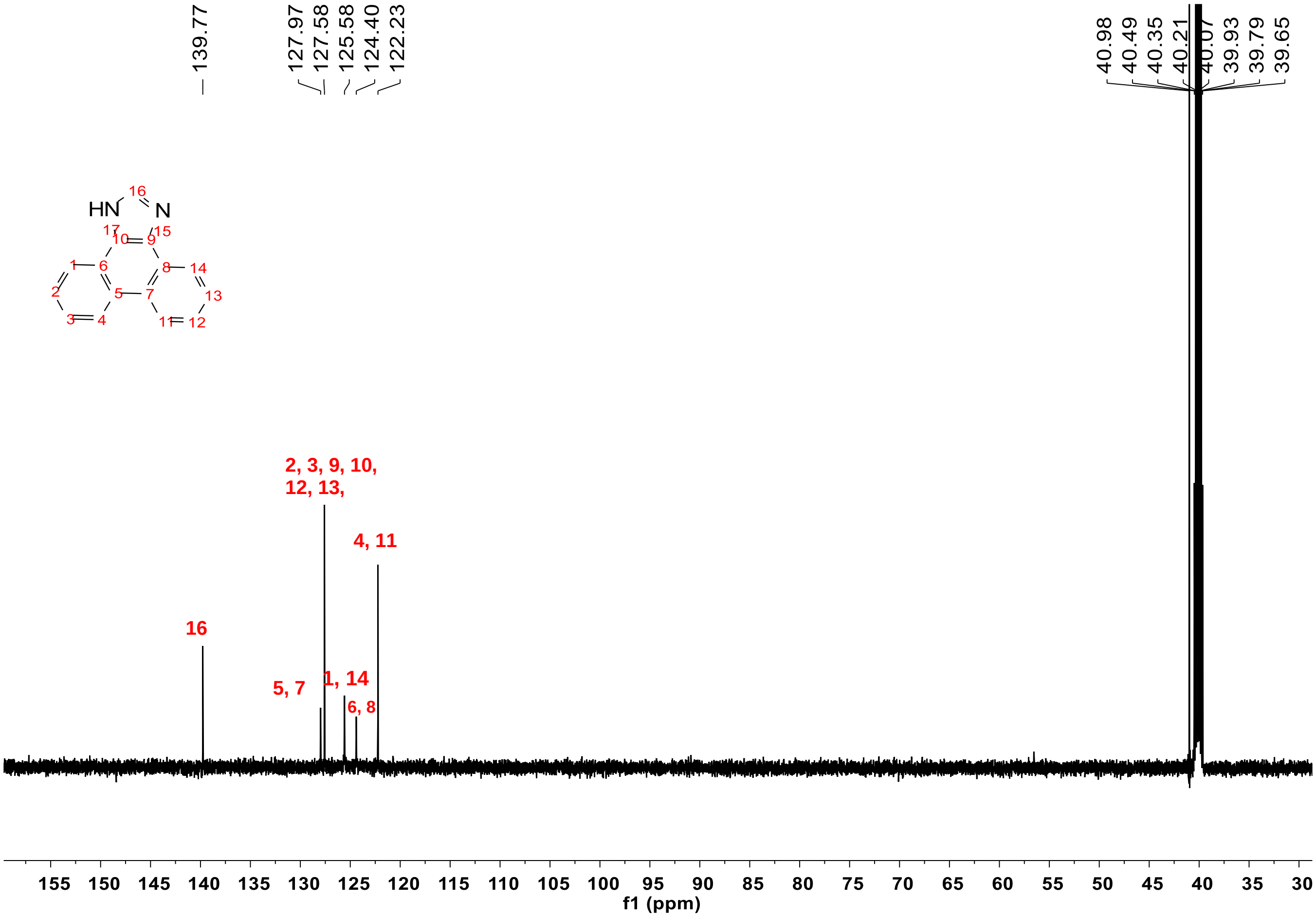

Supplementary Figure 2. ^{13}C NMR spectrum (101 MHz, DMSO-d6) of 1H-phenanthro[9,10-d]imidazole.

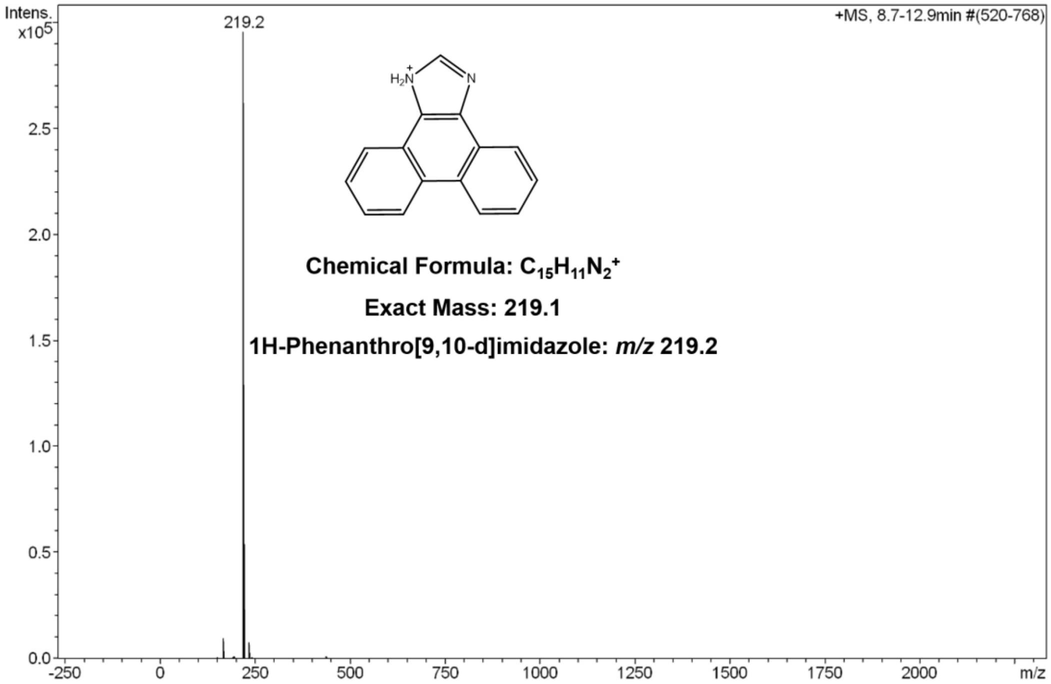

Supplementary Figure 3. ESI-MS spectrum of 1H-phenanthro[9,10-d]imidazole. Calculated for $[C15H11N2]^{+}$: 219.1. Found: 291.2.

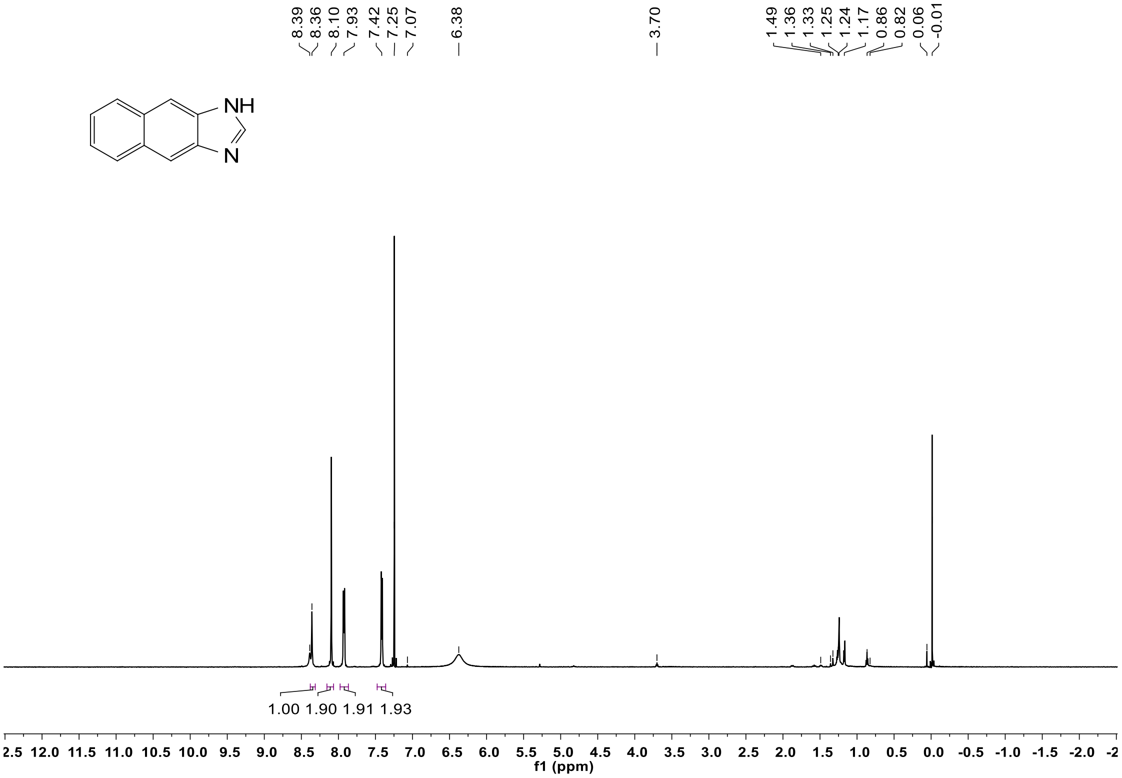

Supplementary Figure 4. ^{1}H NMR spectrum (600 MHz, Chloroform-d) of 1H-naphtho[2,3-d]imidazole.

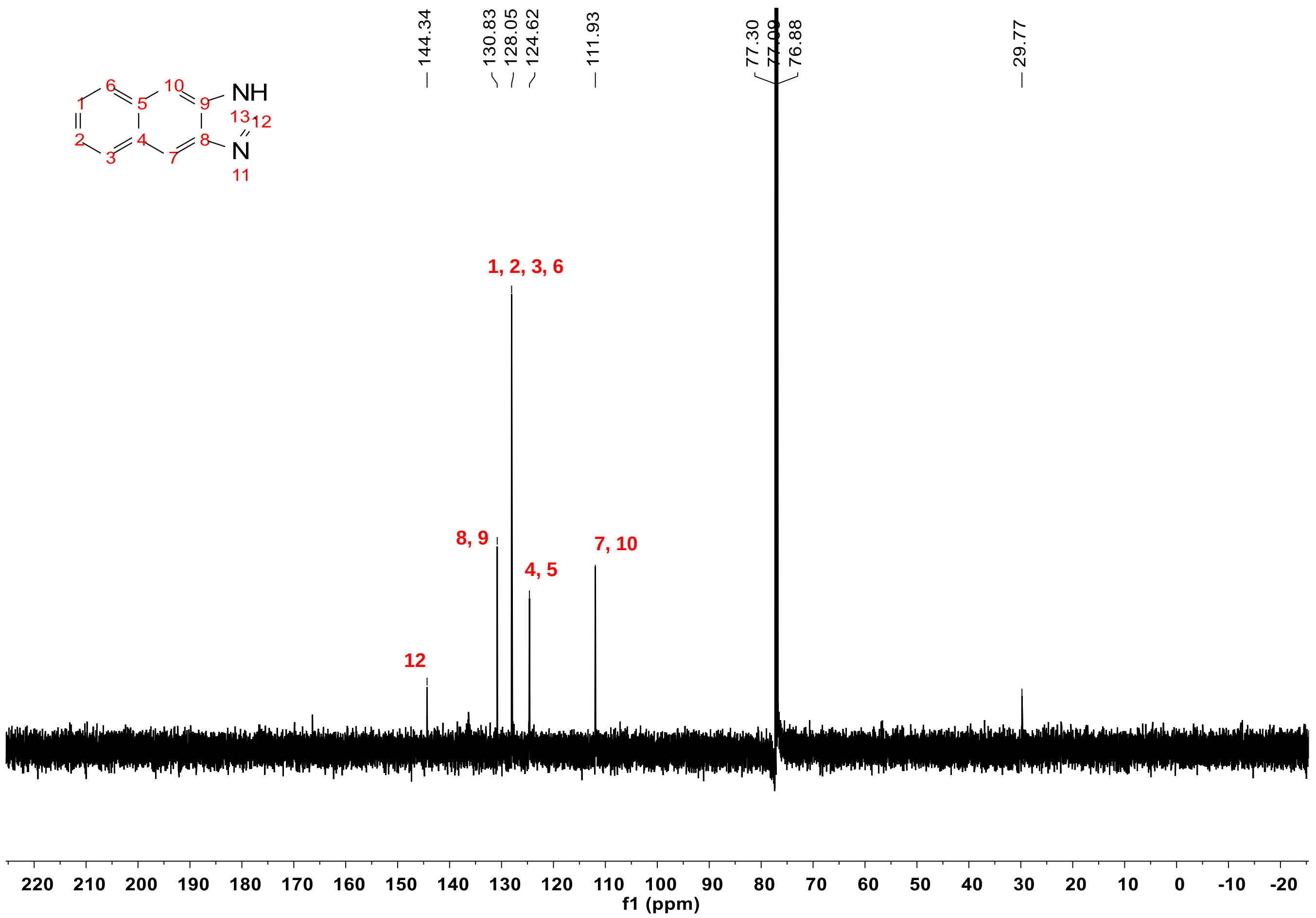

Supplementary Figure 5. ^{1}H NMR spectrum (151 MHz, Chloroform-d) of 1H-naphtho[2,3-d]imidazole.

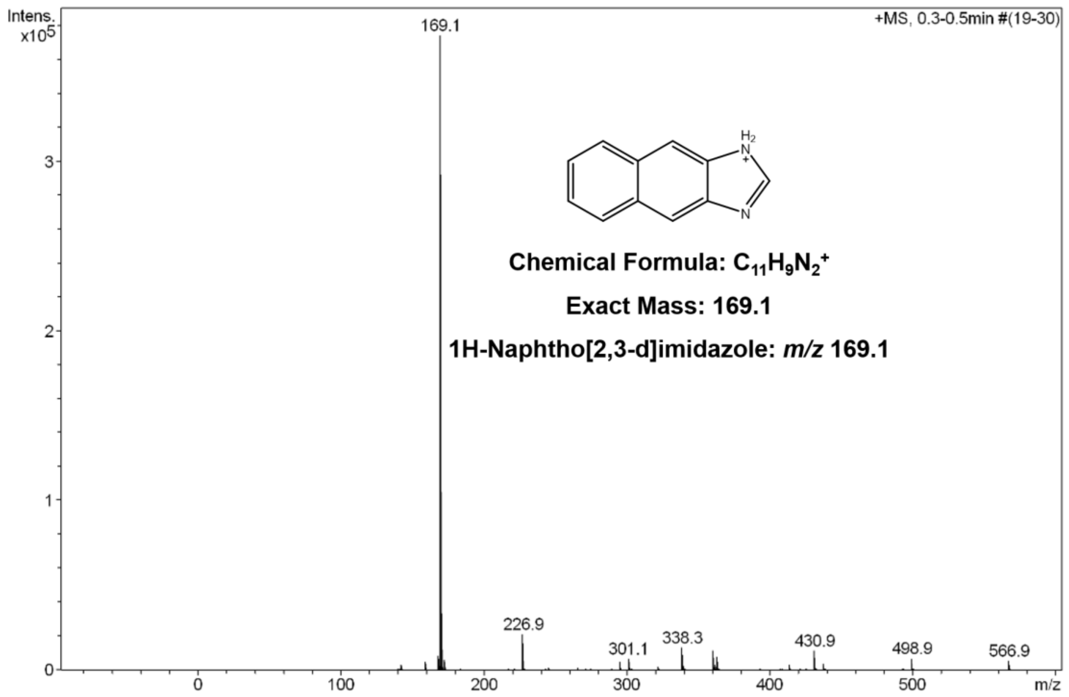

Supplementary Figure 6. ESI-MS spectrum of 1H-naphtho[2,3-d]imidazole. Calculated for $[C11H9N2]^{+}$: 169.1. Found: 169.1.

2. Supplementary Discussion

2.1 Phosphorescent emission/decay mechanism and excellent polymer matrix.

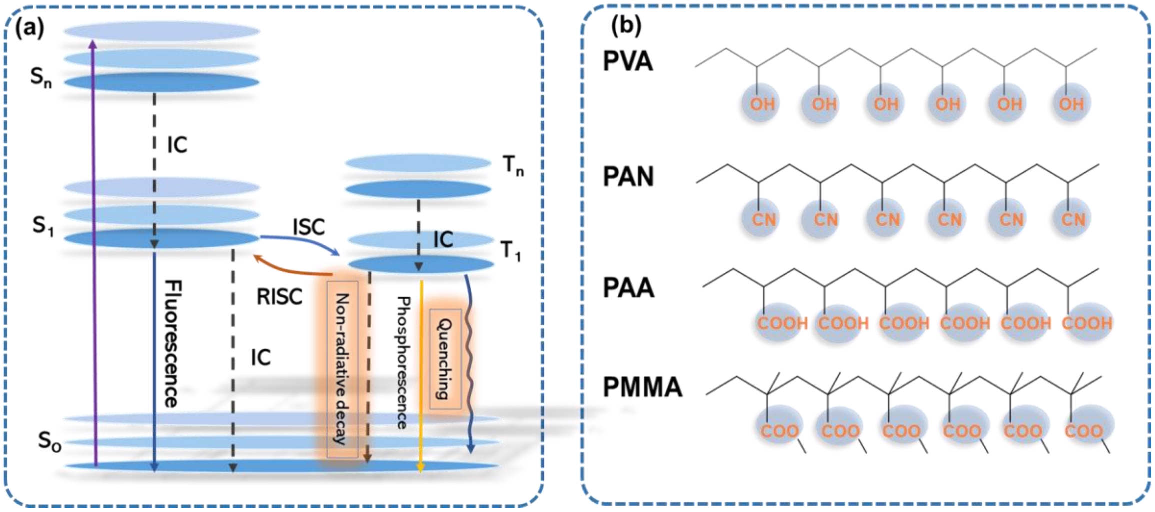

Supplementary Figure 7. (a) Jablonski diagram; **(b)** molecular chains of PMMA, PVA, PAN, and PAA.

2.2 The measurement and characterization of UF-RTPs.

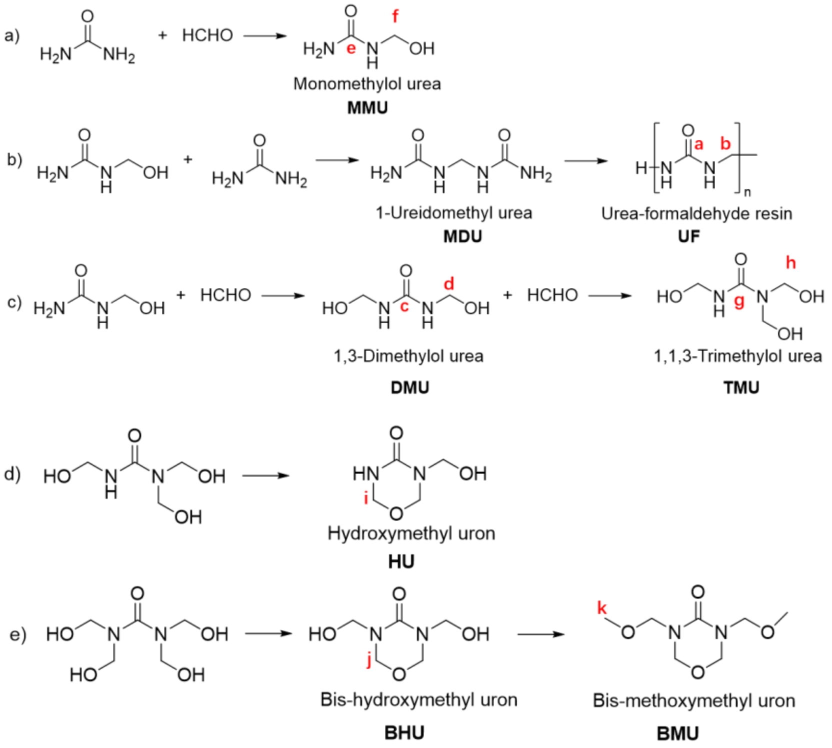

Supplementary Figure 8. Polycondensation reactions of urea with formaldehyde and the formation of ether-bridged intermediates.

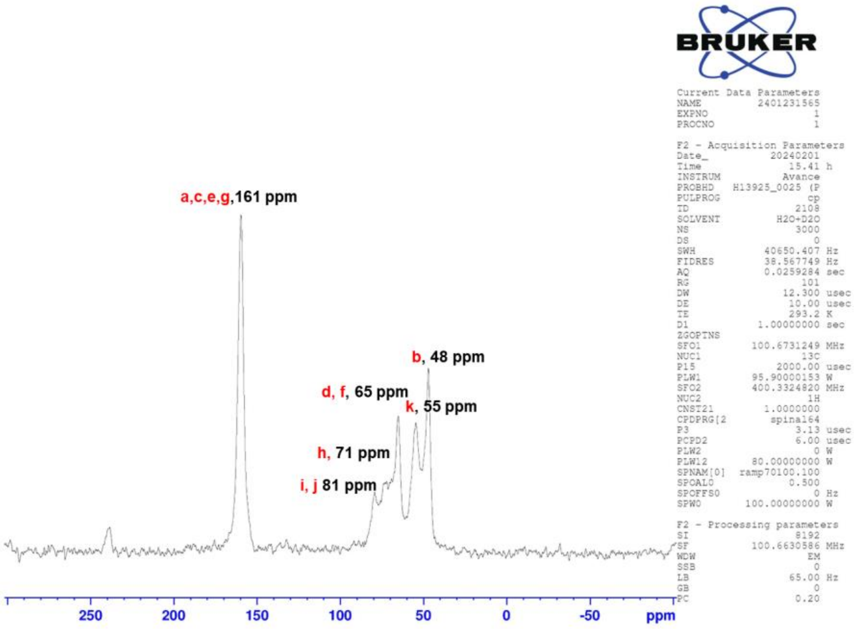

Supplementary Figure 9. ^{13}C-SSNMR spectrum of commercial UF.

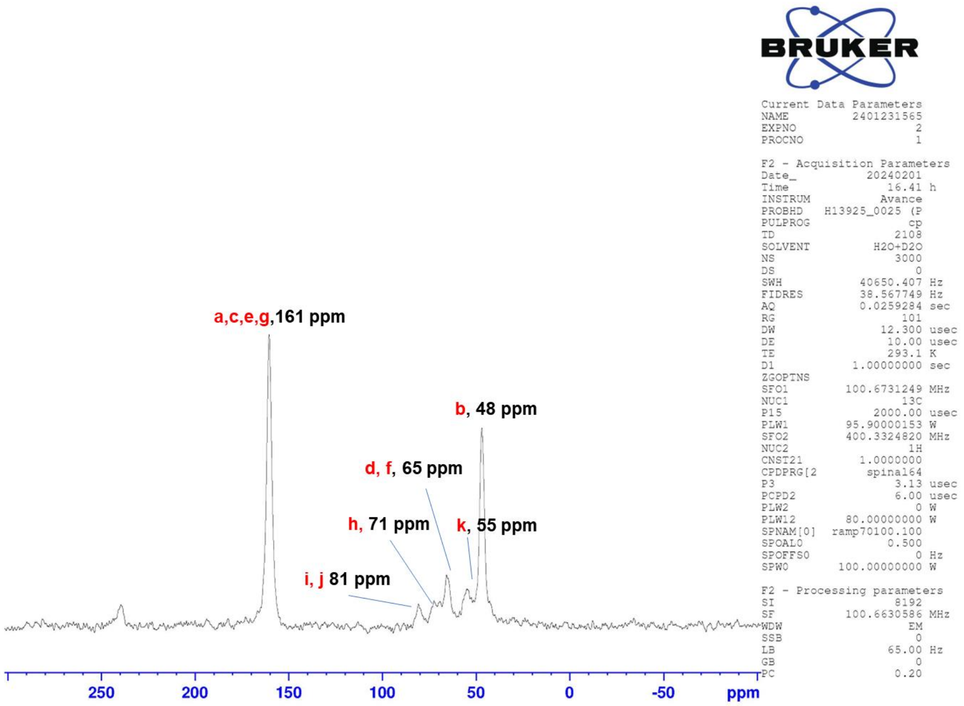

Supplementary Figure 10. ^{13}C-SSNMR spectrum of the prepared UF.

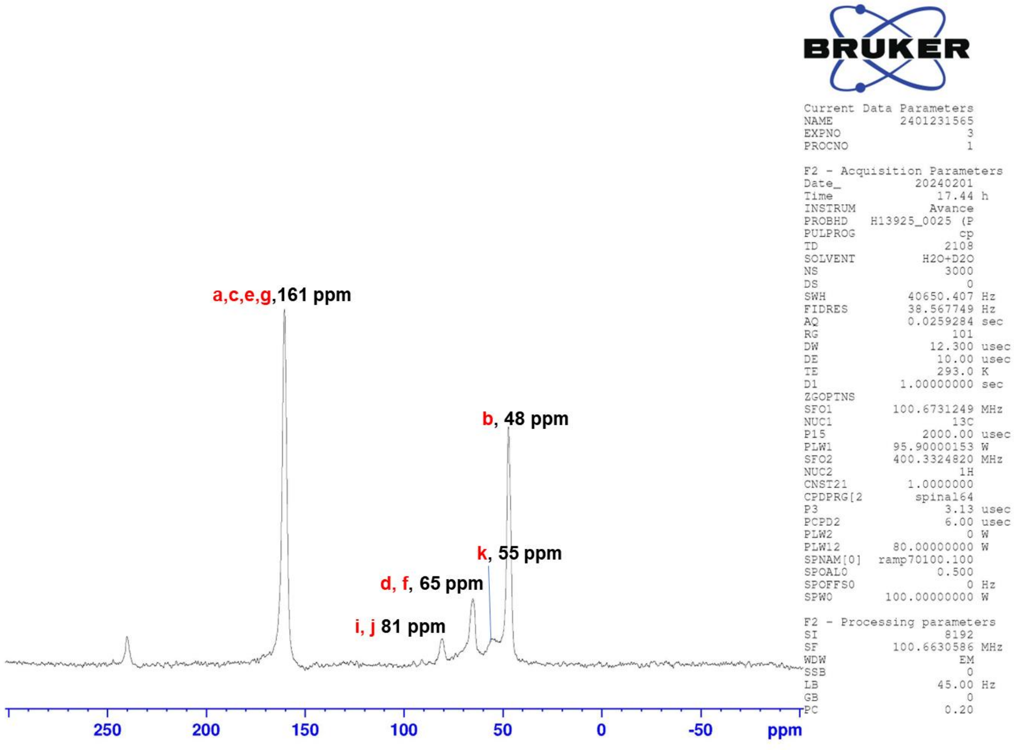

Supplementary Figure 11. ^{13}C-SSNMR spectrum of 14DAP/UF.

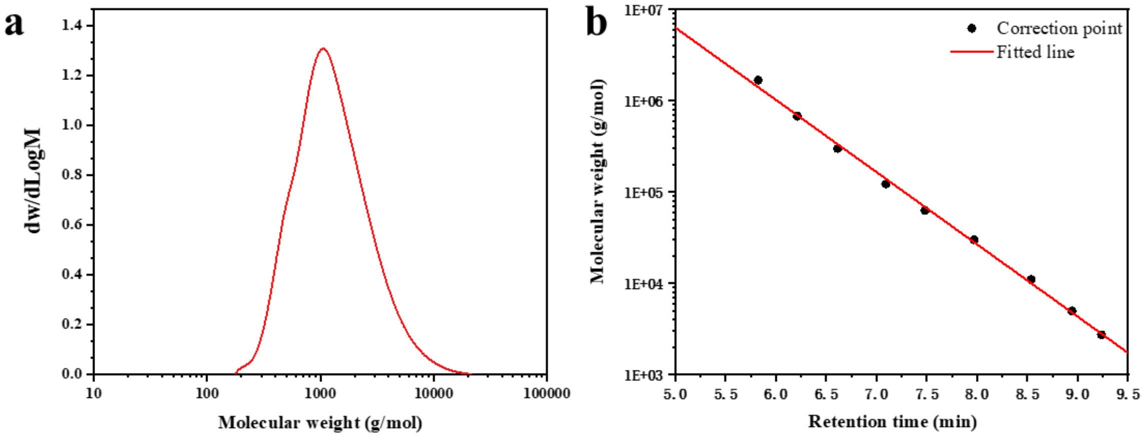

Supplementary Figure 12. (a) GPC spectrum of 14DAP/UF and (b) column calibration plots.

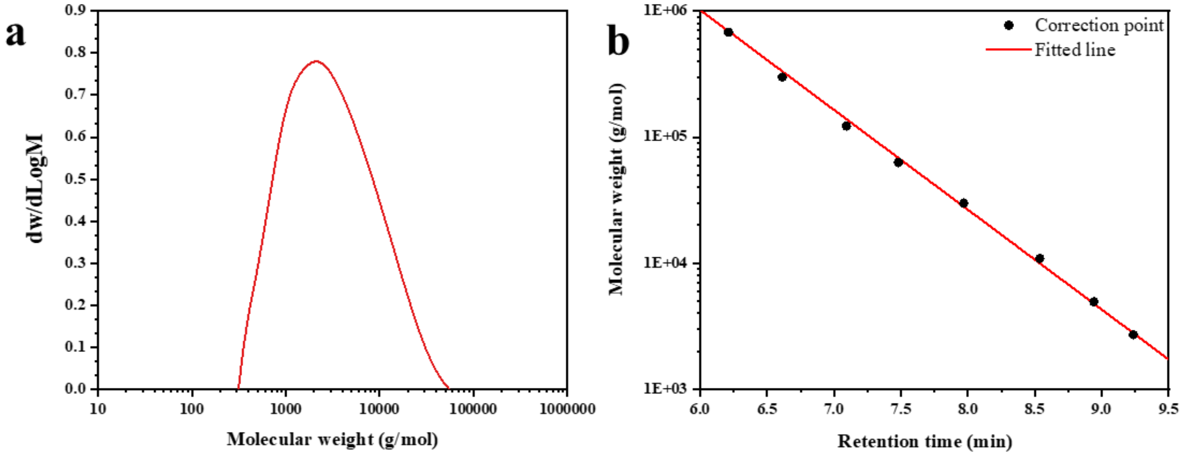

Supplementary Figure 13. (a) GPC spectrum of the commercial UF and (b) column calibration plots.

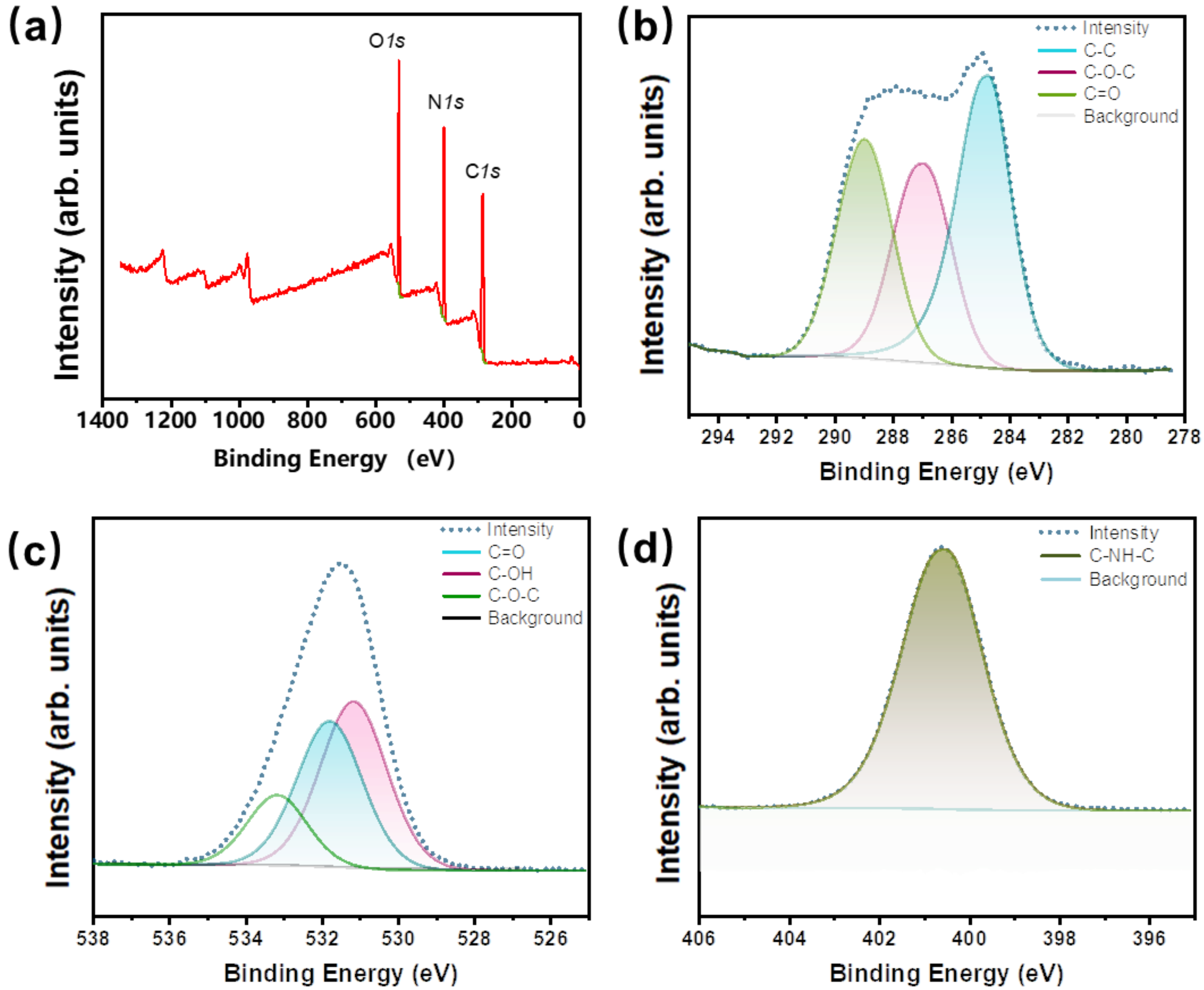

Supplementary Figure 14. XPS spectra of 14DAP/UF (a) XPS full spectrum of 14DAP/UF pyrolyzed at 100 ℃ for 15 min; (b) XPS-C*1s* fine spectrum; (c) XPS-O*1s* fine spectrum; (d) XPS-N*1s* fine spectrum.

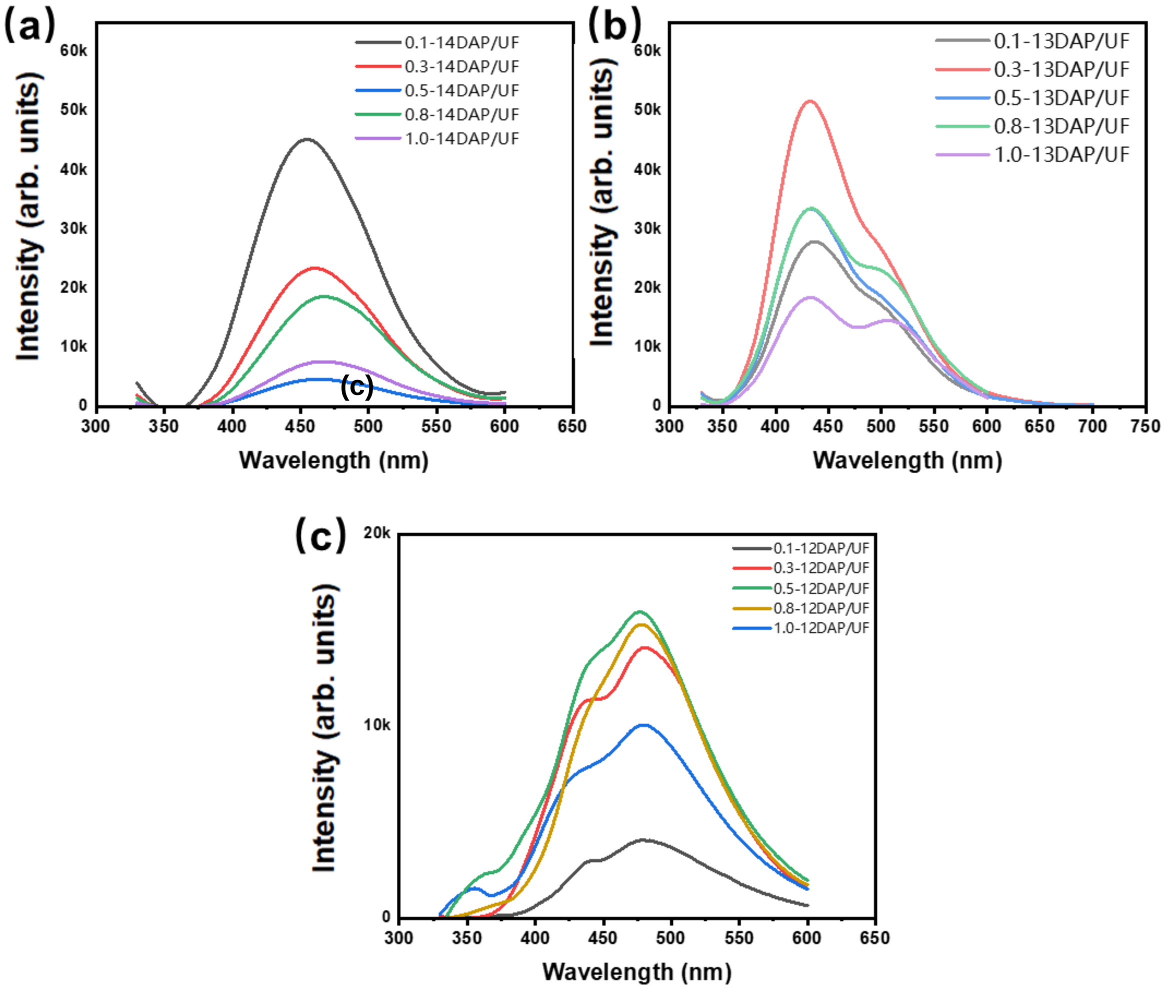

Supplementary Figure 15. Phosphorescence spectra of UF-RTPs prepared with 0.1wt%-1wt% doping concentration of diaminobenzene: (a) 14DAP/UF, (b) 13DAP/UF and (c) 12DAP/UF.

2.3 The measurement and characterization of diaminobenzene derivatives in UF-RTPs.

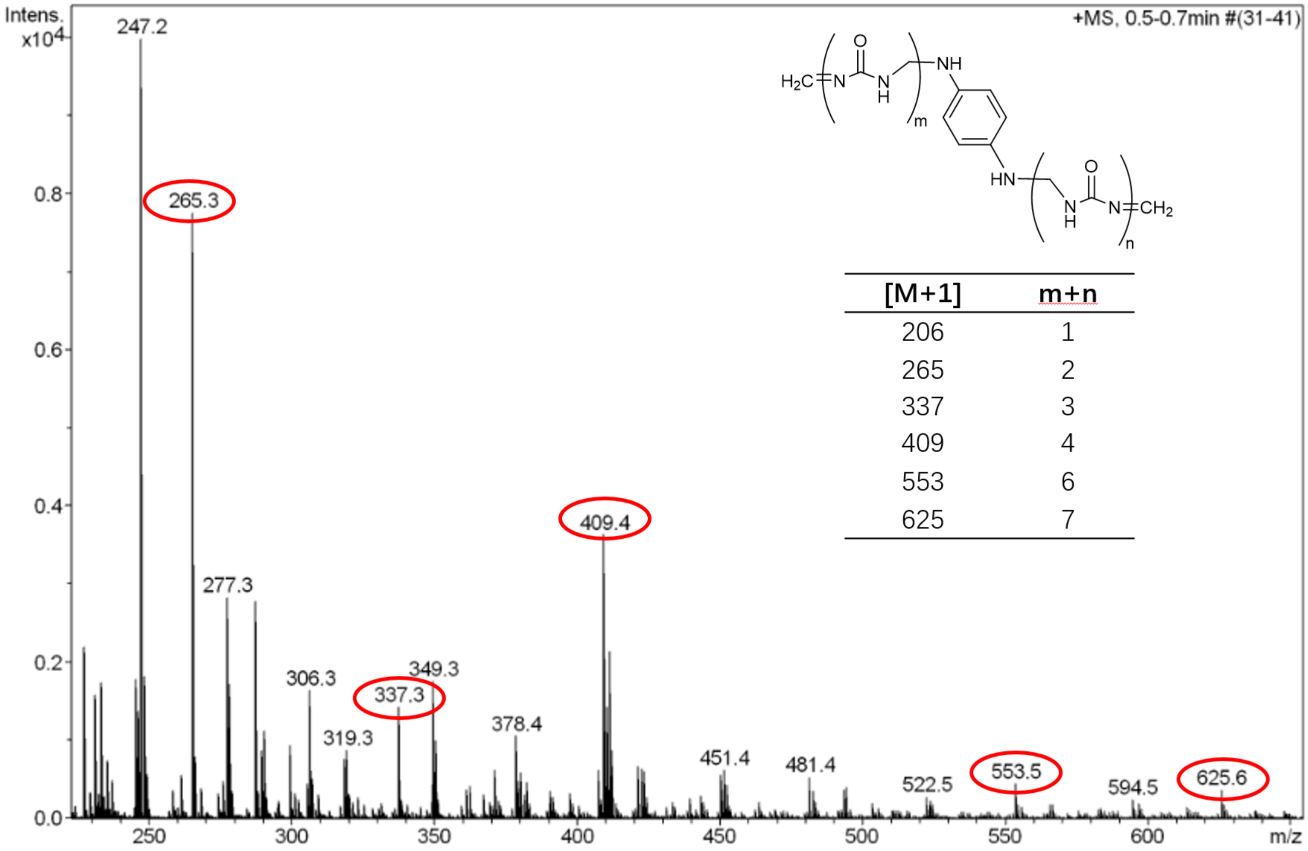

Supplementary Figure 16. ESI-MS spectra of 14DAP/UF extract.

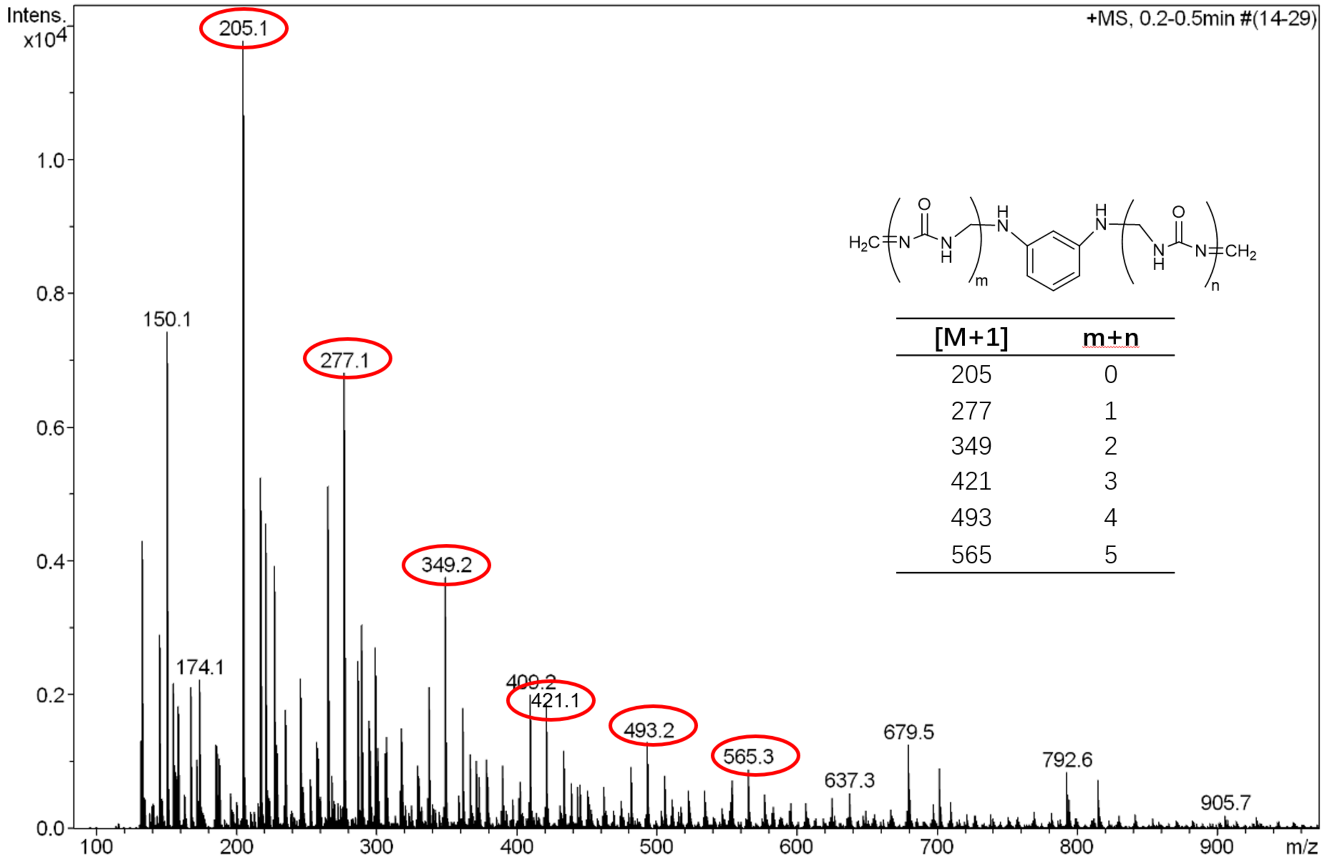

Supplementary Figure 17. ESI-MS spectra of 13DAP/UF extract.

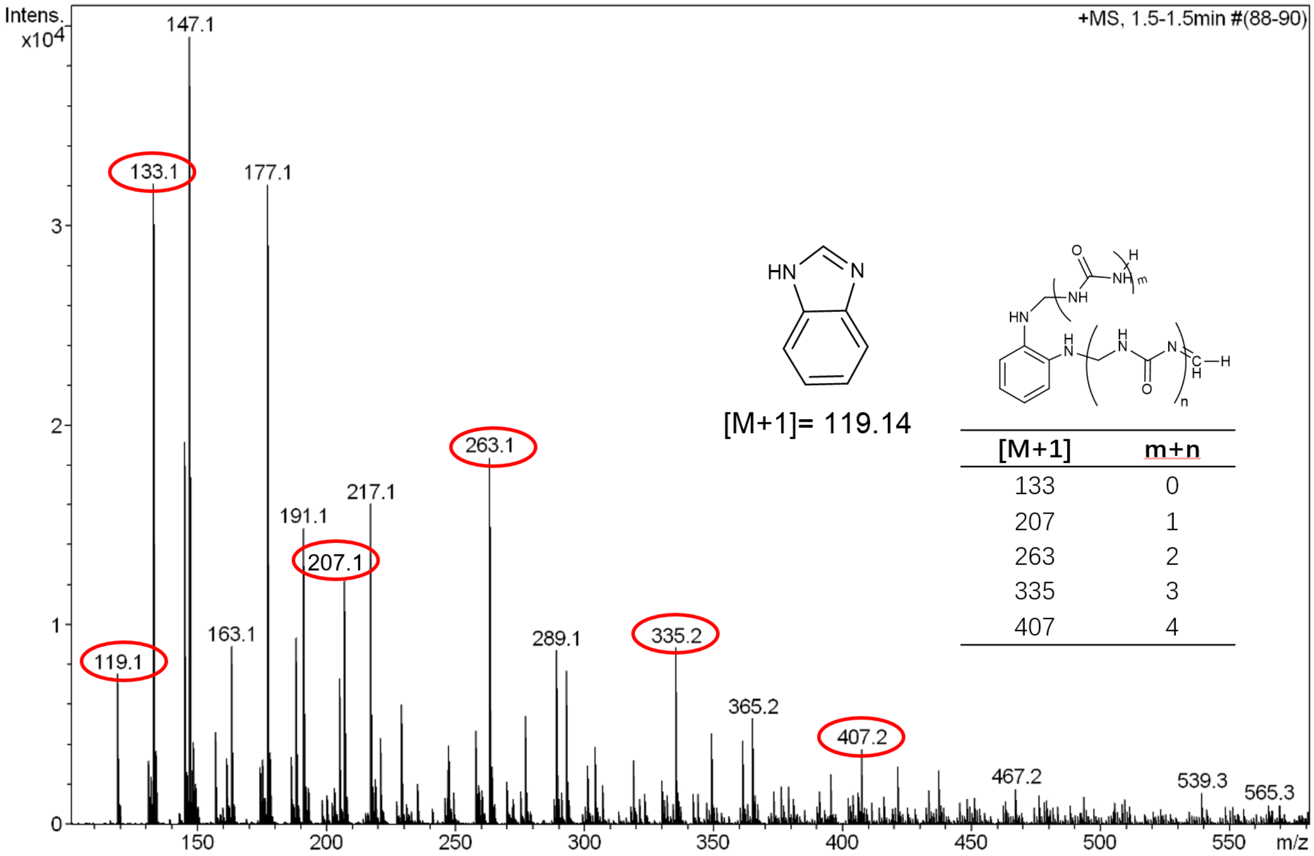

Supplementary Figure 18. ESI-MS spectra of 12DAP/UF extract.

Supplementary Table 1. LC-MS analysis results of UF-RTPs alcoholic extracts.

**[M+1]**			**m+n**
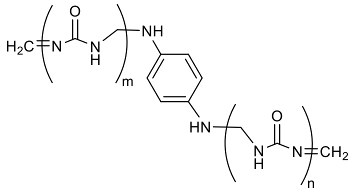	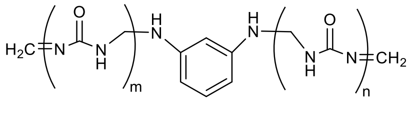	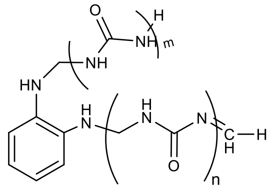	
14DAP/UF	13DAP/UF	12DAP/UF	
-	-	133	0
206	205	207	1
265	277	263	2
337	349	335	3
409	421	407	4
-	493	-	5
553	565	-	6
625	-	-	7

2.4 Analysis of ESP distribution and poles of UF chains and the guest molecules.

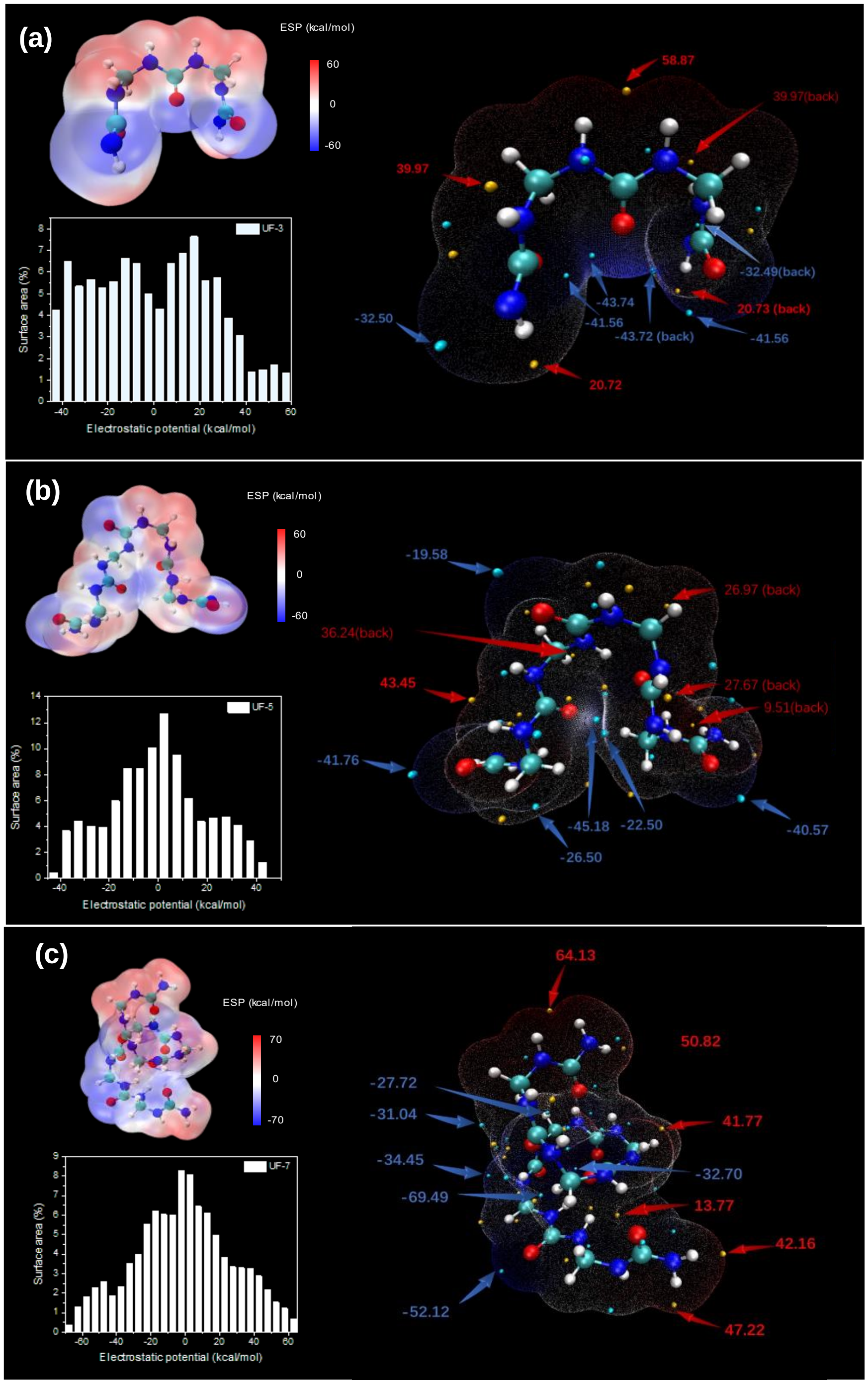

Supplementary Figure 19. The spatial configuration, electrostatic potential energy distribution, and ESP pole distribution of urea formaldehyde chains with (a) 3, (b) 5 and (c) 7 units, respectively.

Supplementary Table 2. The ESP pole distribution of 14DAP and 14DAP-UF.

Molecular	Number	X (Å)	Y (Å)	Z (Å)	kcal/mol	Type
	1	-3.988	-2.015	-0.015	3.44	C
	2	-3.999	1.997	-0.02	3.43	C
	3	1.837	3.643	-0.038	35.9	C
	4	1.862	-3.64	-0.026	35.9	C
	5	3.576	0.008	0.034	50.31	C
**14DAP**	1	-1.257	0.009	-1.873	-29.39	O
	2	-1.25	0.012	1.871	-29.39	O
	3	1.174	-1.238	-1.899	-19.96	O
	4	1.215	-1.241	1.904	-19.97	O
	5	1.242	1.226	1.907	-19.95	O
	6	1.174	1.242	-1.899	-19.97	O
	1	-6.117	-2.381	2.679	37.77	C
	2	-5.695	1.5	-2.281	33.58	C
	3	-3.955	0.183	2.793	42.54	C
	4	-3.112	-2.148	-0.492	24.43	C
	5	-3.136	3.289	-0.424	19.1	C
	6	-2.718	3.193	0.074	19.16	C
	7	2.218	-2.711	-0.407	12.63	C
	8	3.222	2.266	1.542	35.81	C
	9	5.39	0.001	2.936	49.13	C
	10	6.897	1.498	2.123	48	C
**14DAP-UF**	11	8.885	0.474	-0.725	38.08	C
	1	-8.229	-1.471	-0.985	-42.7	O
	2	-6.482	1.863	0.458	-14.7	O
	3	-4.48	-2.649	0.451	-12.16	O
	4	-2.656	0.144	-2.584	-28.11	O
	5	-0.275	-0.491	1.464	-16.84	O
	6	0.303	0.223	-2.149	-25.29	O
	7	0.336	1.103	-1.885	-24.68	O
	8	3.33	0.95	-1.658	-34.48	O
	9	5.177	-0.864	-2.838	-42.02	O
	10	7.68	-1.72	1.093	-17.54	O

The type of C and O represent maximum and minimum points respectively.

Supplementary Table 3. The ESP pole distribution of 13DAP and 13DAP-UF.

Molecular	Number	X (Å)	Y (Å)	Z (Å)	kcal/mol	Type
	1	-4.431	-0.066	-0.017	34.22	C
	2	-2.533	-3.209	-0.013	35.14	C
	3	-0.001	4.242	-0.019	4.39	C
	4	2.549	-3.208	-0.012	35.14	C
	5	4.43	-0.064	-0.031	34.21	C
**13DAP**	1	-2.001	-0.683	-1.904	-21.32	O
	2	-1.98	-0.683	1.902	-21.31	O
	3	0.004	0.823	-1.831	-29.47	O
	4	-0.03	0.879	1.85	-29.46	O
	5	1.991	-0.683	1.903	-21.32	O
	6	2.001	-0.683	-1.904	-21.32	O
	1	-4.651	-0.755	-2.329	9.92	C
	2	-3.356	-2.641	0.485	-8.79	C
	3	-2.334	-0.187	2.622	42.77	C
	4	-1.057	4.544	2.827	13.69	C
	5	-0.66	-0.465	-2.737	23.23	C
	6	-0.349	-3.447	0.605	-7.39	C
	7	1.894	-1.285	-2.982	40.99	C
	8	2.944	-3.165	0.039	-7.51	C
	9	4.01	0.133	1.507	37.55	C
	10	4.231	1.947	1.246	41.15	C
**13DAP-UF**	1	-4.377	-1.762	0.651	-19.11	O
	2	-2.314	-3.733	1.607	-28.47	O
	3	-2.367	2.12	-2.732	-28.07	O
	4	-2.072	-3.589	-1.248	-35.08	O
	5	-0.497	3.626	-1.018	-19.2	O
	6	0.355	0.097	1.038	-13.72	O
	7	0.4	0.879	1.781	-13.55	O
	8	1.544	-4.019	-0.637	-25.56	O
	9	1.736	-3.647	2.149	-36.08	O
	10	2.754	2.754	-1.658	-17.89	O
	11	4.037	-2.573	-0.236	-16.26	O

The type of C and O represent maximum and minimum points respectively.

Supplementary Table 4. The ESP pole distribution of 12DAP and 12DAP-UF.

Molecular	Number	X (Å)	Y (Å)	Z (Å)	kcal/mol	Type
	1	-3.988	-2.015	-0.015	3.44	C
	2	-3.999	1.997	-0.02	3.43	C
	3	1.837	3.643	-0.038	35.9	C
	4	1.862	-3.64	-0.026	35.9	C
	5	3.576	0.008	0.034	50.31	C
**12DAP**	1	-1.257	0.009	-1.873	-29.39	O
	2	-1.25	0.012	1.871	-29.39	O
	3	1.174	-1.238	-1.899	-19.96	O
	4	1.215	-1.241	1.904	-19.97	O
	5	1.242	1.226	1.907	-19.95	O
	6	1.174	1.242	-1.899	-19.97	O
	1	-6.602	0.224	0.655	11.84	C
	2	-5.43	-3.616	0.349	11.59	C
	3	-0.934	1.85	2.781	9.24	C
	4	-0.5	0.373	2.11	9.45	C
	5	0.172	-3.786	-0.547	42.33	C
	6	0.191	4.879	-1.493	45.11	C
	7	0.903	1.682	-1.438	9.55	C
	8	1.861	5.758	-0.609	48.67	C
	9	3.003	0.383	-0.615	-3.06	C
	10	3.148	-2.416	1.798	-16.63	C
	11	3.276	-5.166	-1.027	29.14	C
**12DAP-UF**	12	4.594	3.482	-0.158	39.08	C
	1	-3.765	-0.265	-1.853	-18.09	O
	2	-3.162	-1.09	1.895	-15.84	O
	3	-1.7	1.632	-2.338	-25.91	O
	4	0.173	4.363	1.655	-7.46	O
	5	1.059	-0.446	-1.802	-23.18	O
	6	2.494	2.813	-2.281	-13.67	O
	7	2.716	1.213	1.974	-29.38	O
	8	2.981	-3.847	1.866	-28.7	O
	9	3.158	-1.067	1.957	-25.73	O
	10	5.635	-1.327	-0.318	-49.97	O

The type of C and O represent maximum and minimum points respectively.

2.5 Quantum chemical calculations of guest molecules.

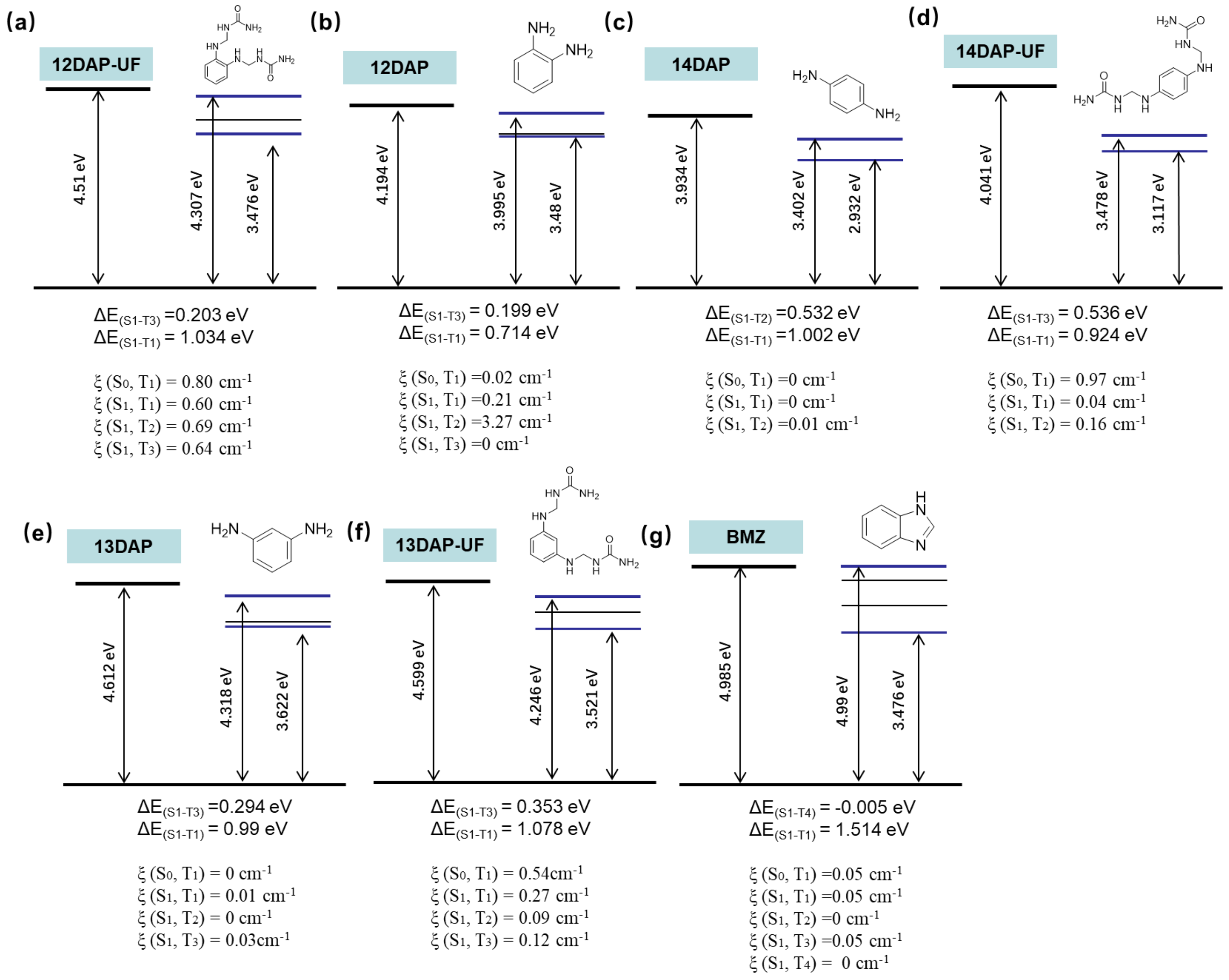

Supplementary Figure 20.: The energy levels and SOC of (a)-(g) guest molecules.

2.6 The photophysical property of UF-RTPs.

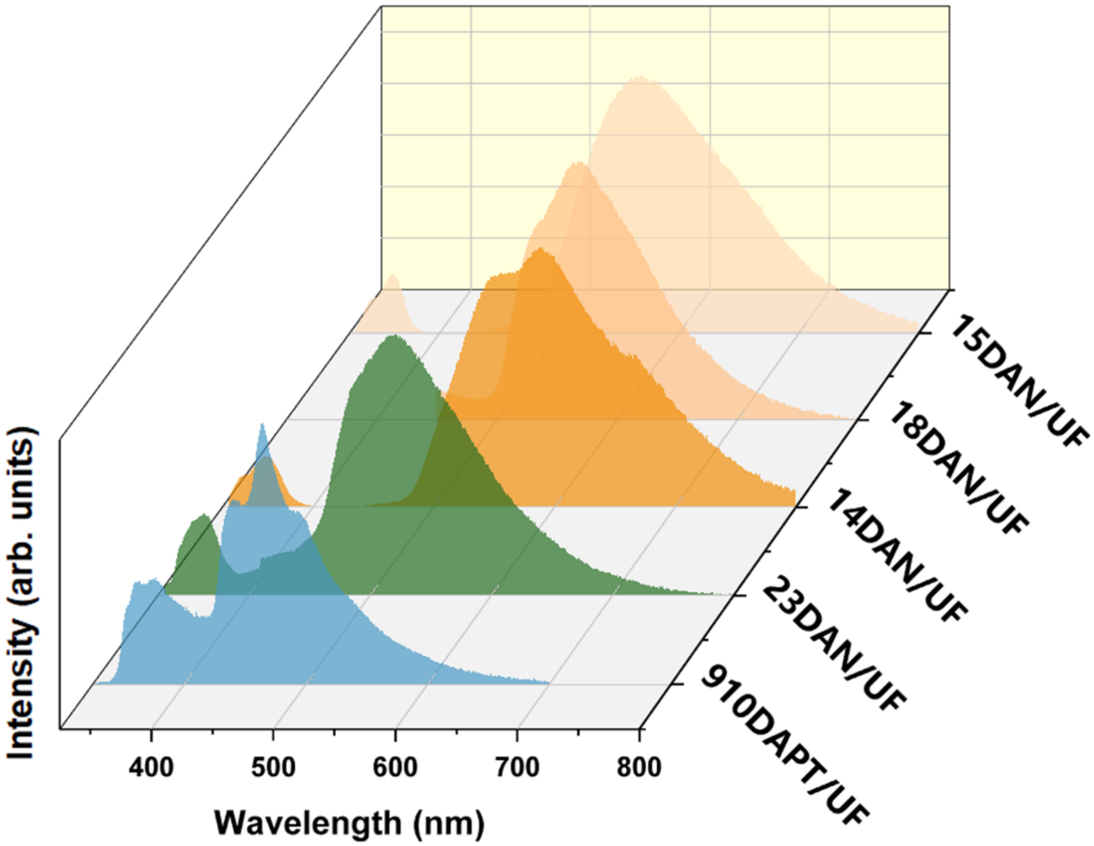

Supplementary Figure 21. Phosphorescence spectra of DAN/UFs and 910DAPT/UF.

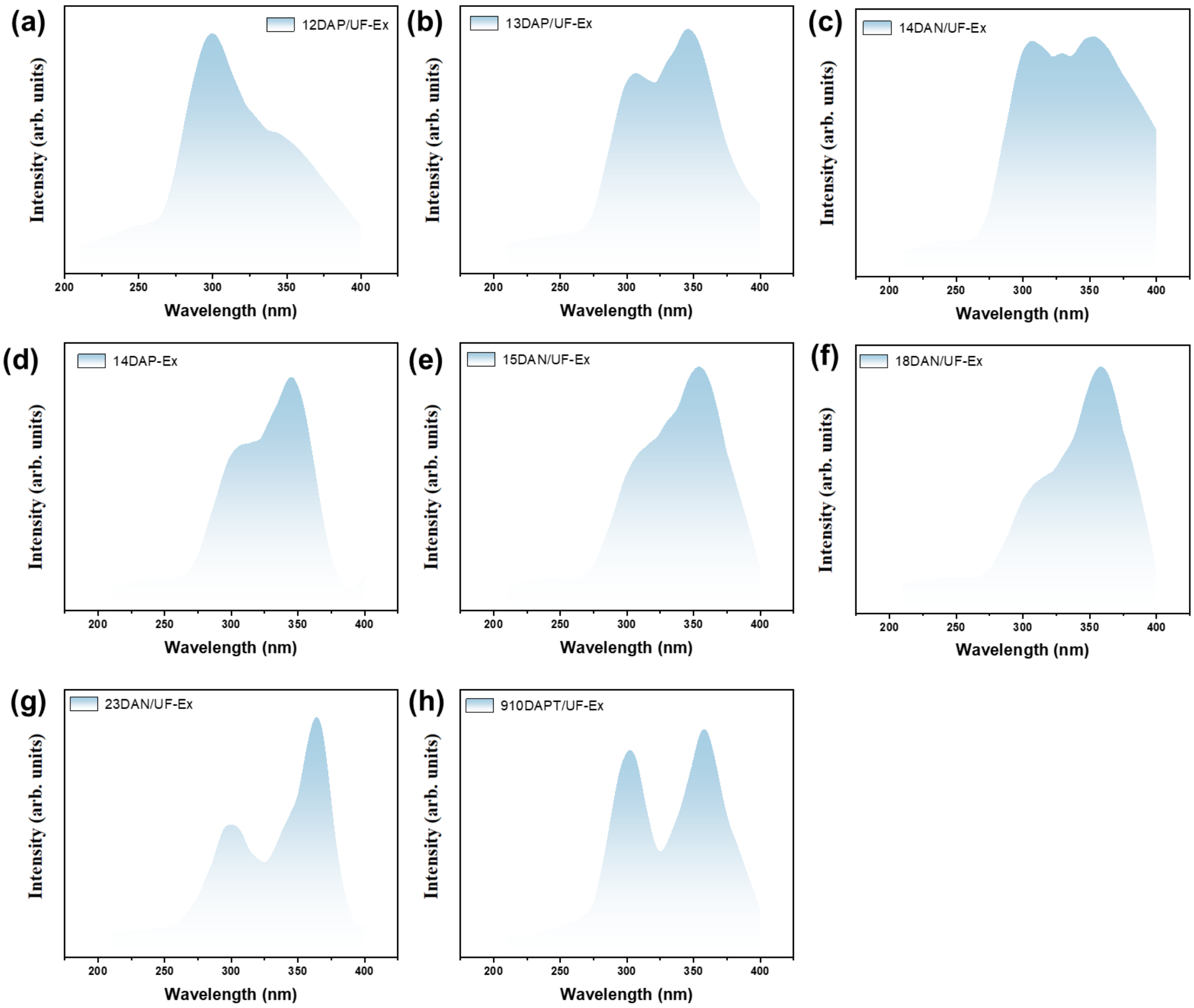

Supplementary Figure 22. Excitation spectra of UF-RTPs.

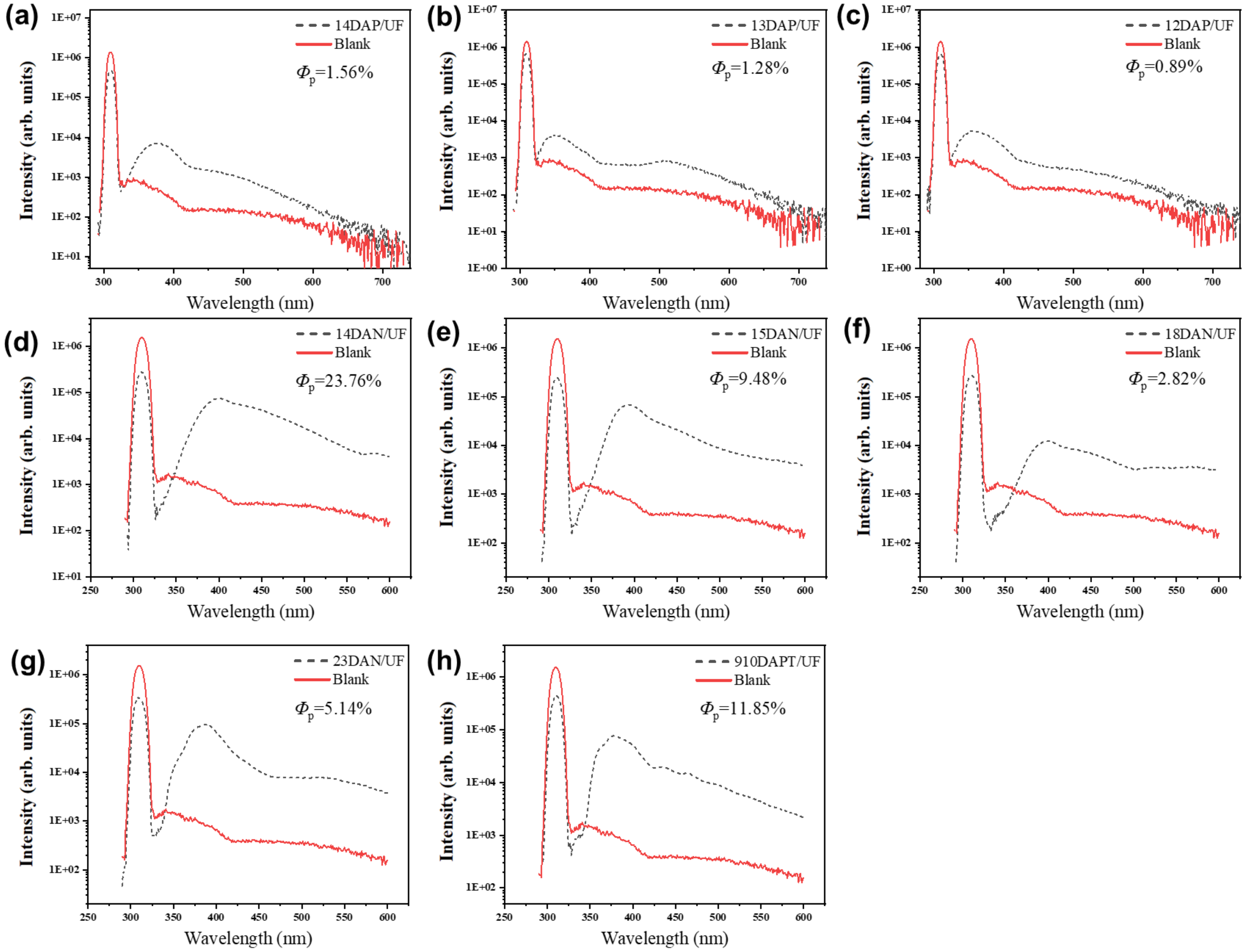

Supplementary Figure 23. Photoluminescence and phosphorescence spectra and quantum yield of (a)-(h) UF-RTPs.

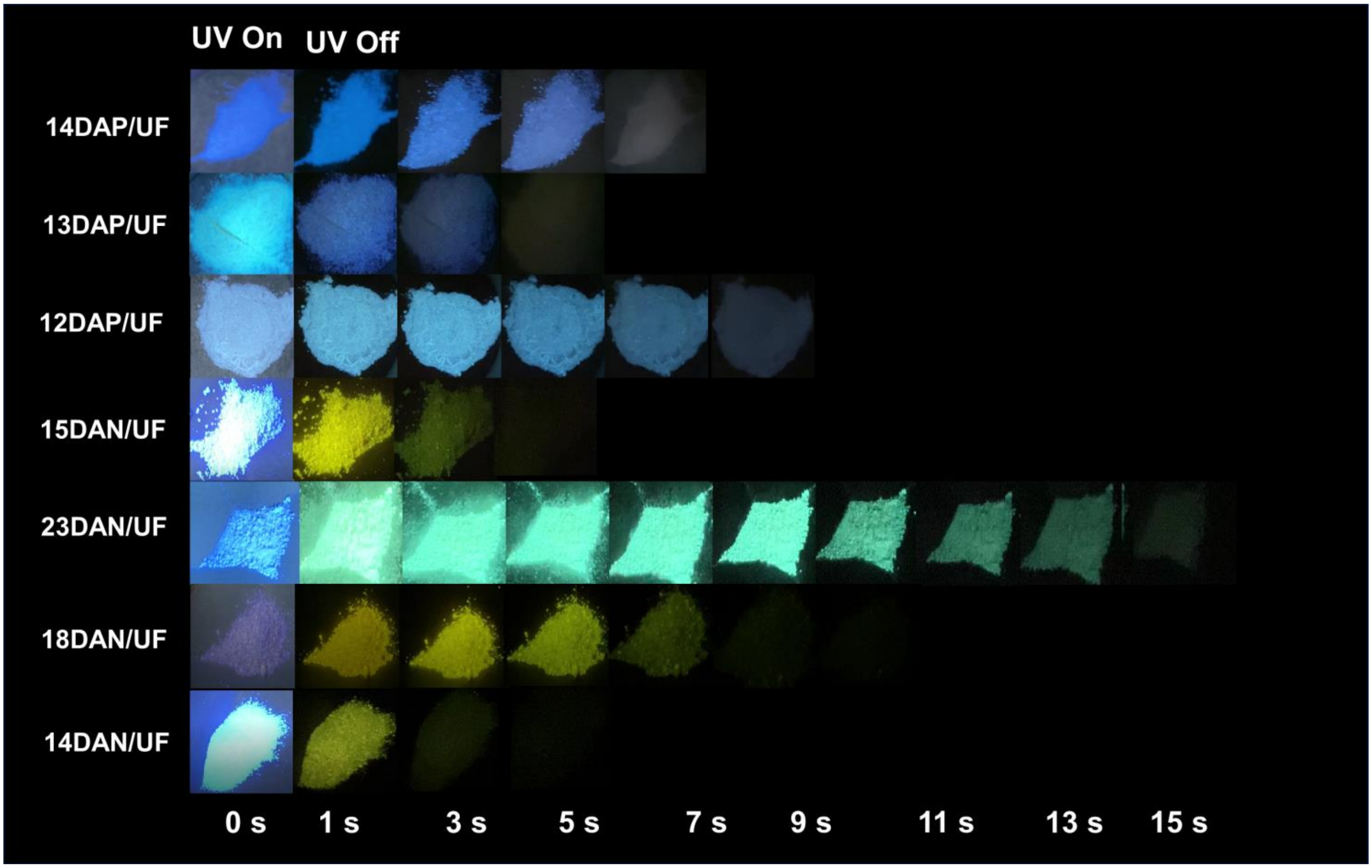

Supplementary Figure 24 Photographs of DAP/UFs and DAN/UFs.

2.7 Thes luminescence mechanism of aromatic *o*-diamine in UF-RTPs.

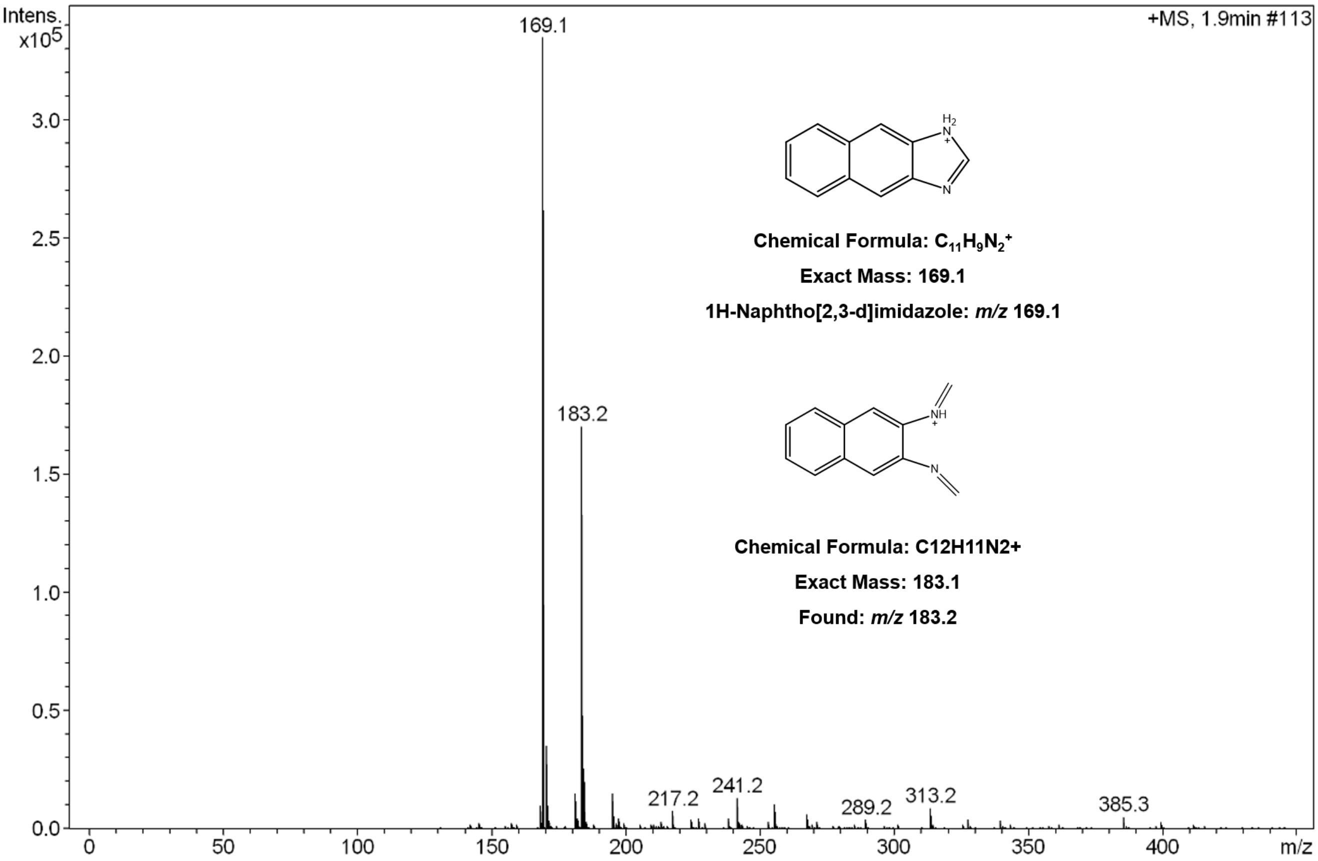

Supplementary Figure 25. ESI-MS spectrum of 23DAN/UF extract. Calculated for $[C11H9N2]^{+}$: 169.1, and $[C12H11N2]^{+}$: 183.1. Found: 169.1, and 183.2.

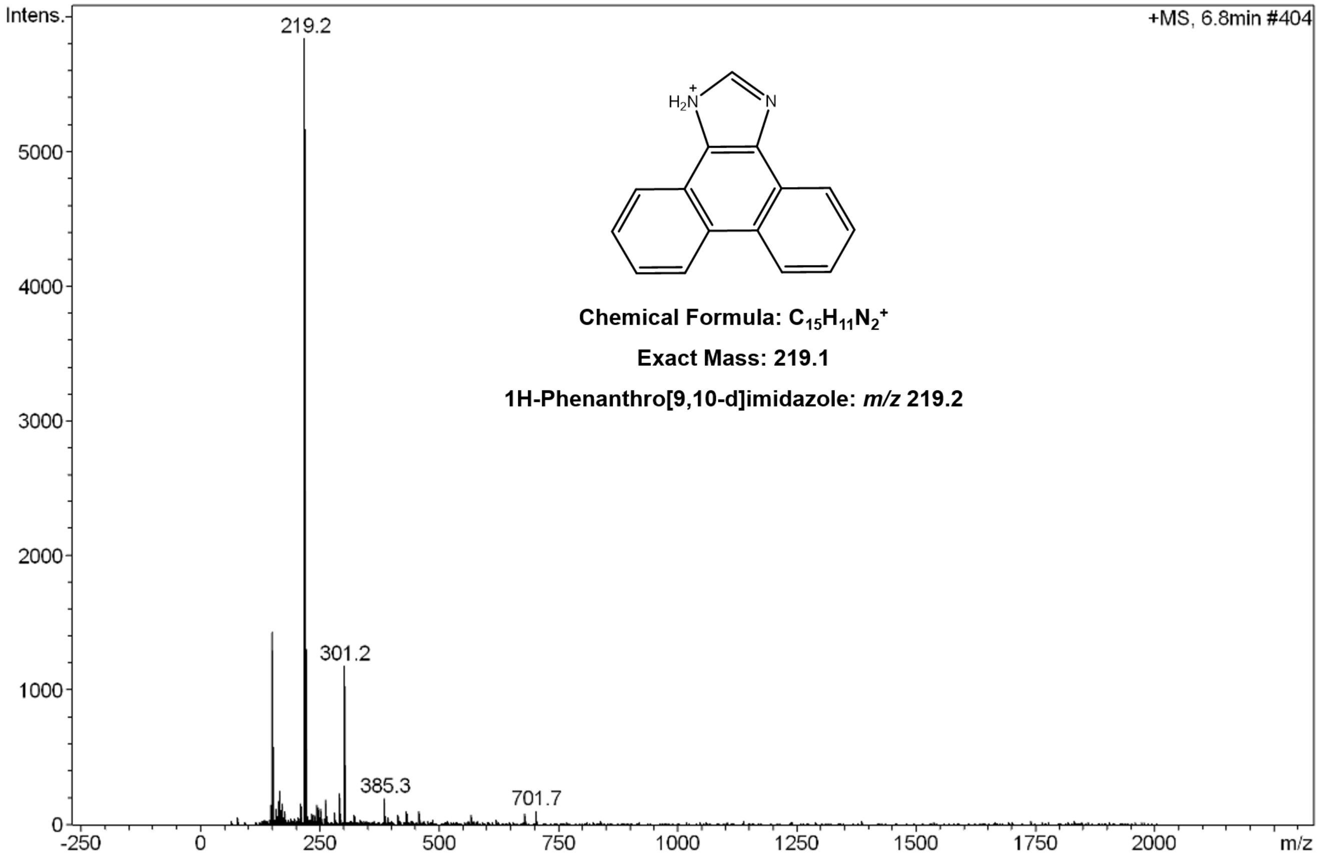

Supplementary Figure 26. ESI-MS spectrum of 910DAPT/UF extract. Calculated for $[C15H11N2]^{+}$: 219.1. Found: 219.2.

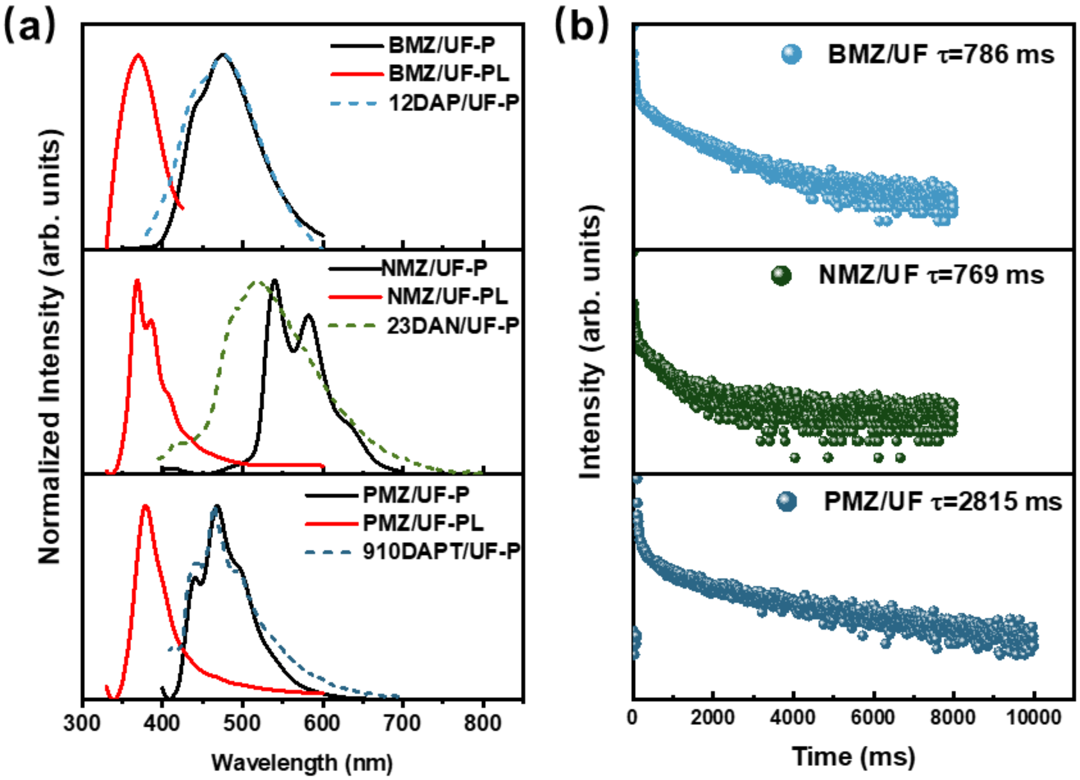

Supplementary Figure 27. RTP performance of aromatic *o*-diamines doped UF resin and the phosphorescent light source: (a) Photoluminescence and phosphorescence spectra of 12DAP/UF, BMZ/UF, 23DAN/UF, NMZ/UF, 910DAPT/UF and PMZ/UF; (b) phosphorescence decay lifetimes of BMZ/UF, NMZ/UF and PMZ/UF.

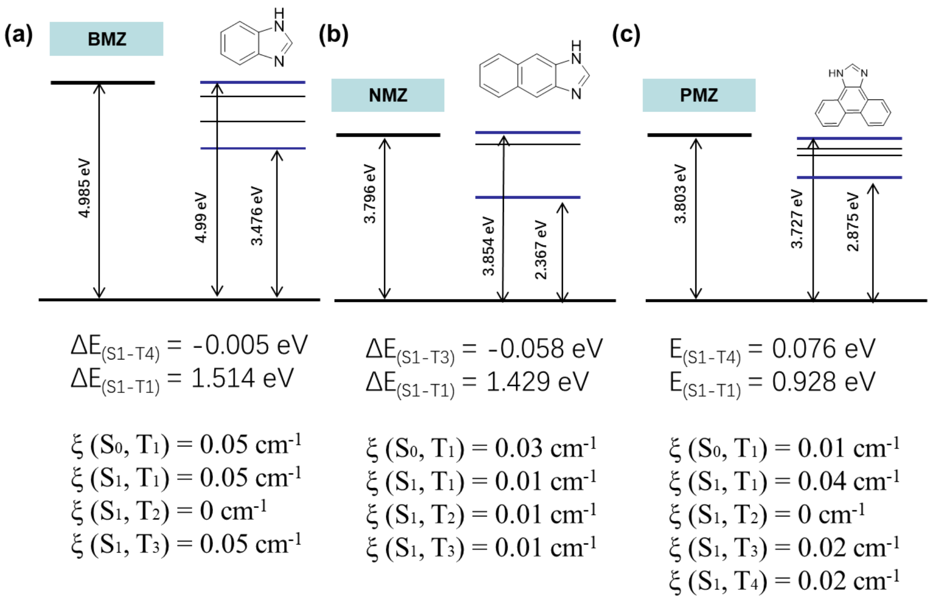

Supplementary Figure 28. Calculation of energy level differences for BMZ, NMZ, and PMZ: ΔE_{ST} of (a) BMZ, (b) NMZ, and (c) PMZ.

2.8 Stability of UF-RTPs.

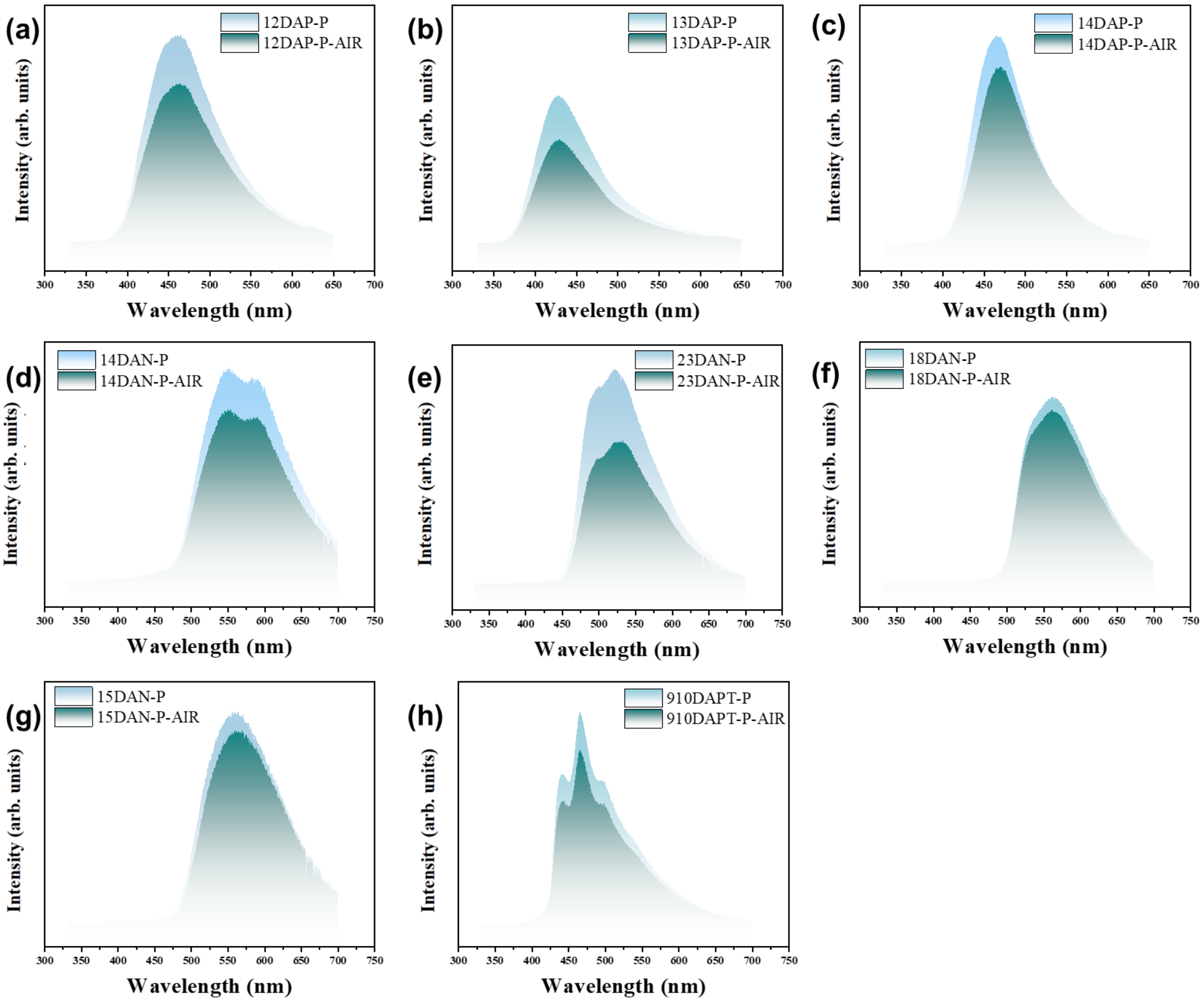

Supplementary Figure 29. Phosphorescence spectra of (a)-(h) UF-RTPs in air and N_2 atmospheres.

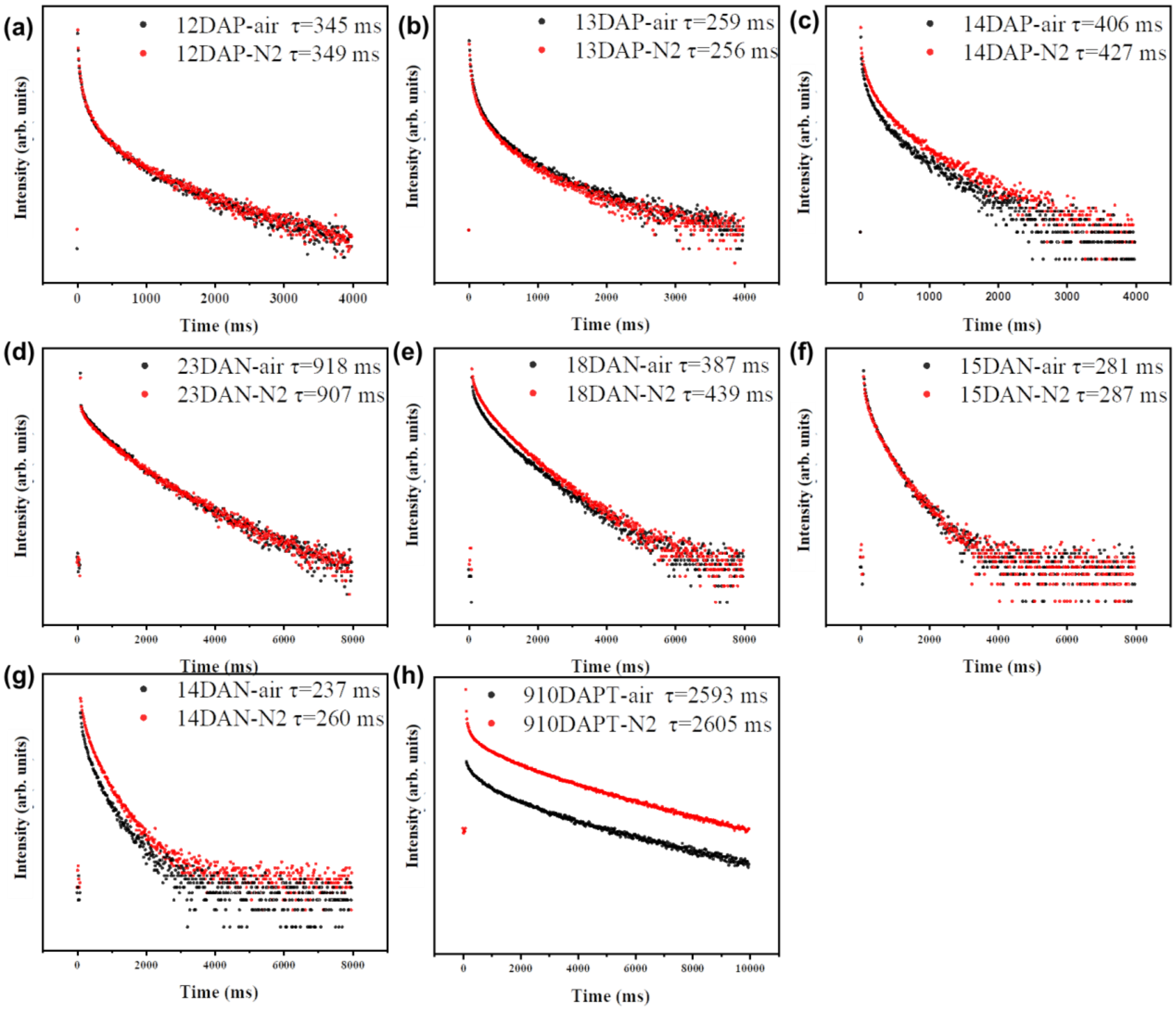

Supplementary Figure 30. Phosphor decay life of (a)-(h) UF-RTPs in air and N_2 atmospheres.

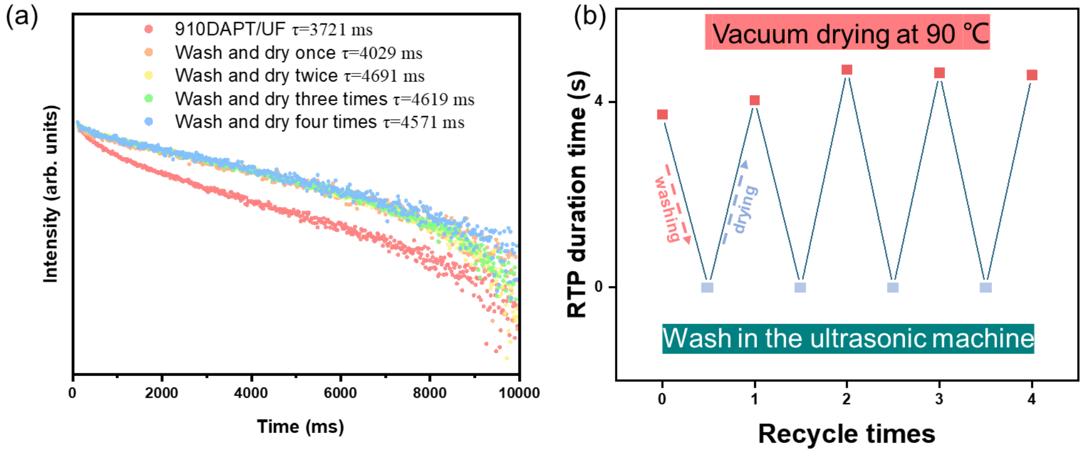

Supplementary Figure 31. The phosphor decay life of 910DAPT/UF during (b) four cycles of washing and drying.

2.9 The measurement and characterization of μUFs.

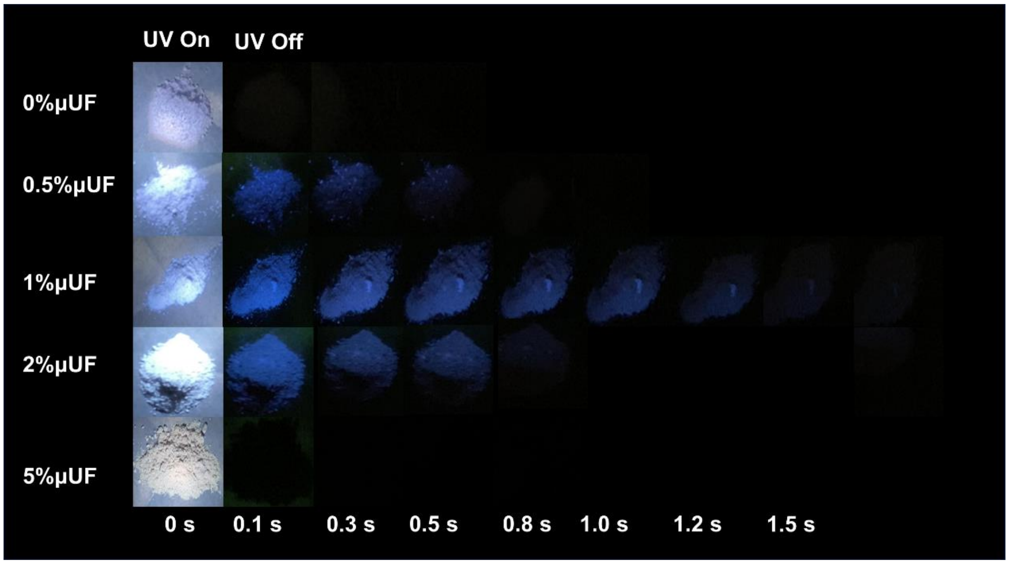

Supplementary Figure 32. Photographs of 0% μUF-5%μUF.

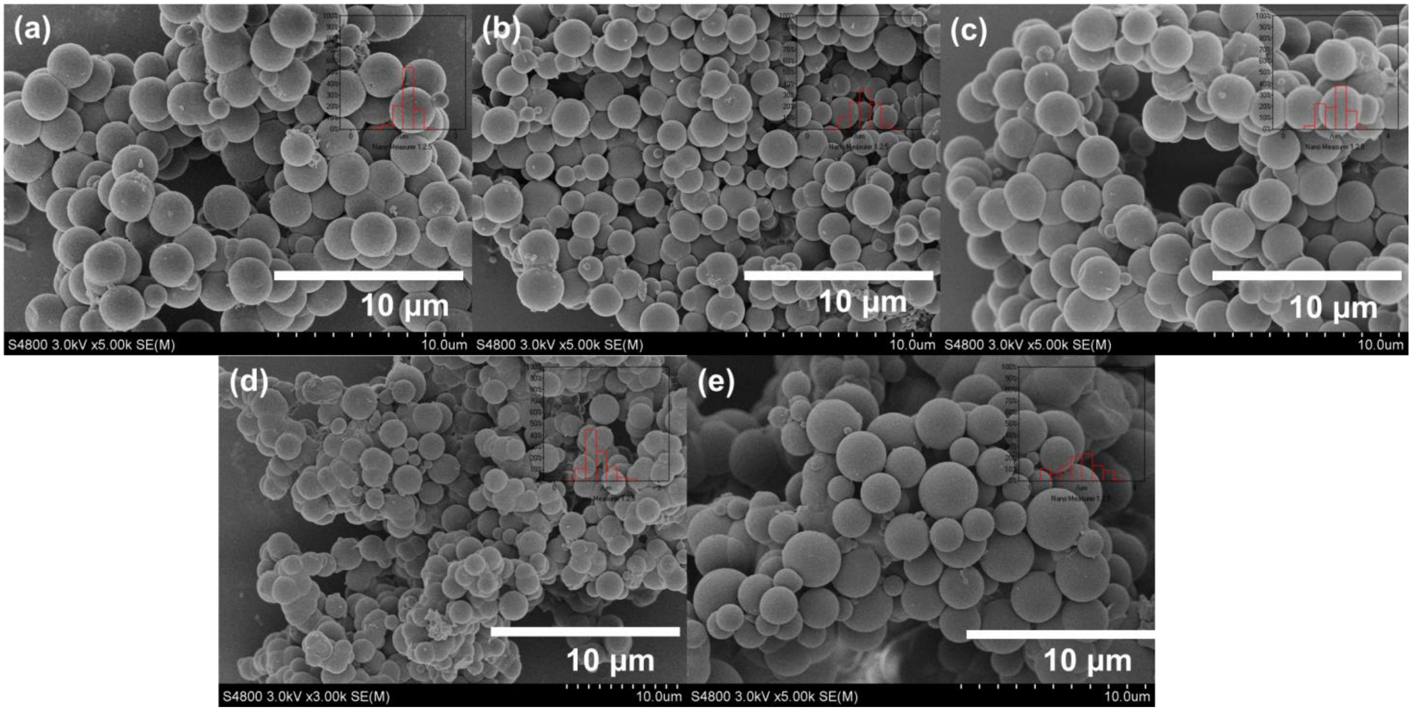

Supplementary Figure 33. SEM images and particle size distribution statistics: (a) 0% μUF, (b) 0.5% μUF, (c) 1% μUF, (d) 2% μUF, and (e) 5% μUF. Five different locations were selected for each sample, and then statistical analysis of particle size was performed on over 100 microspheres from each sample.

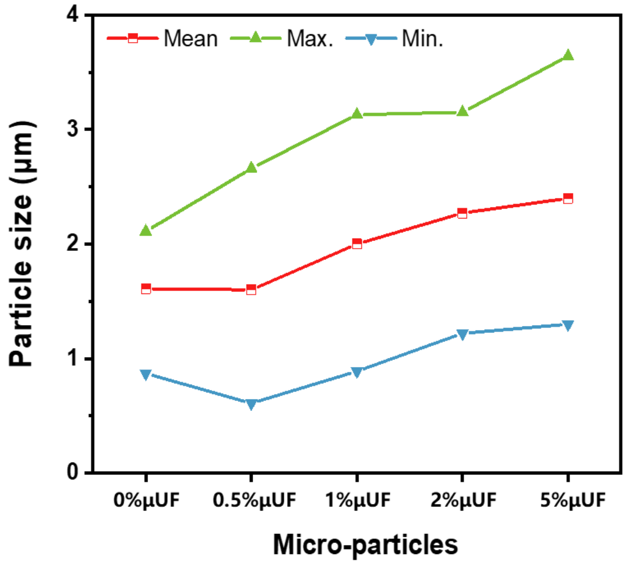

Supplementary Figure 34. Particle size of 0%μUF-5%μUF

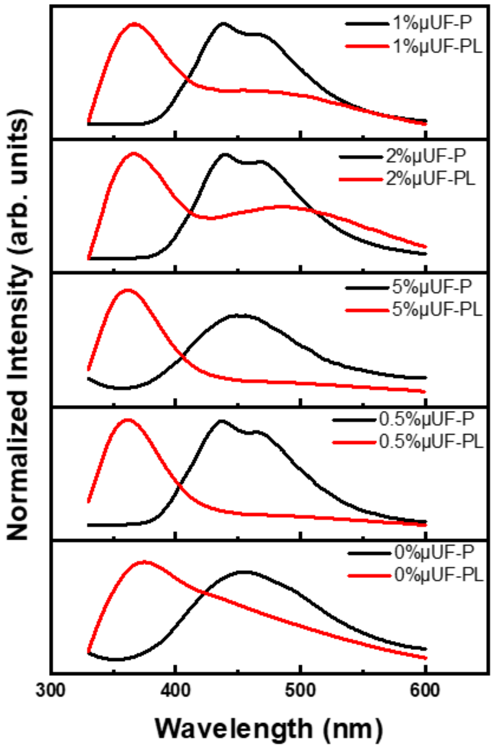

Supplementary Figure 35. Steady-state PL and phosphorescence spectra of 0% μUF, 0.5% μUF, 1% μUF, 2% μUF and 5% μUF.

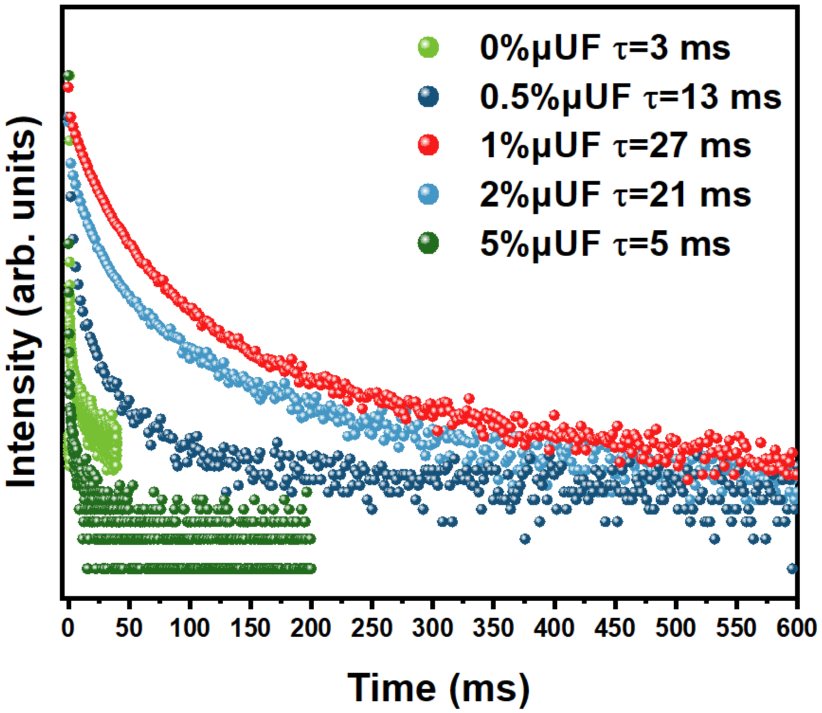

Supplementary Figure 36. Phosphorescence lifetime decay curves of 0% μUF-5% μUF.

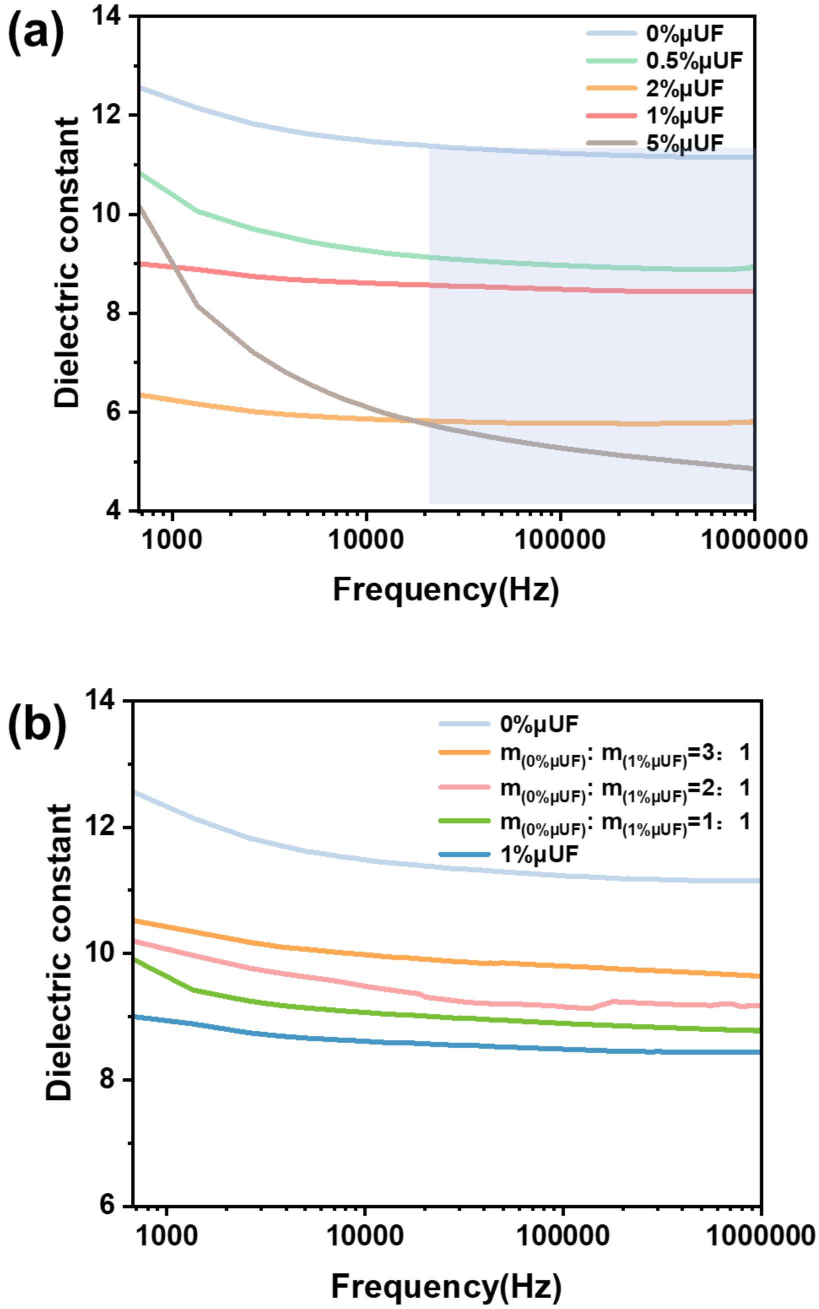

Supplementary Figure 37. Dielectric properties of (a) µUFs and (b) mixtures of 0%µUF and 1%µUF.

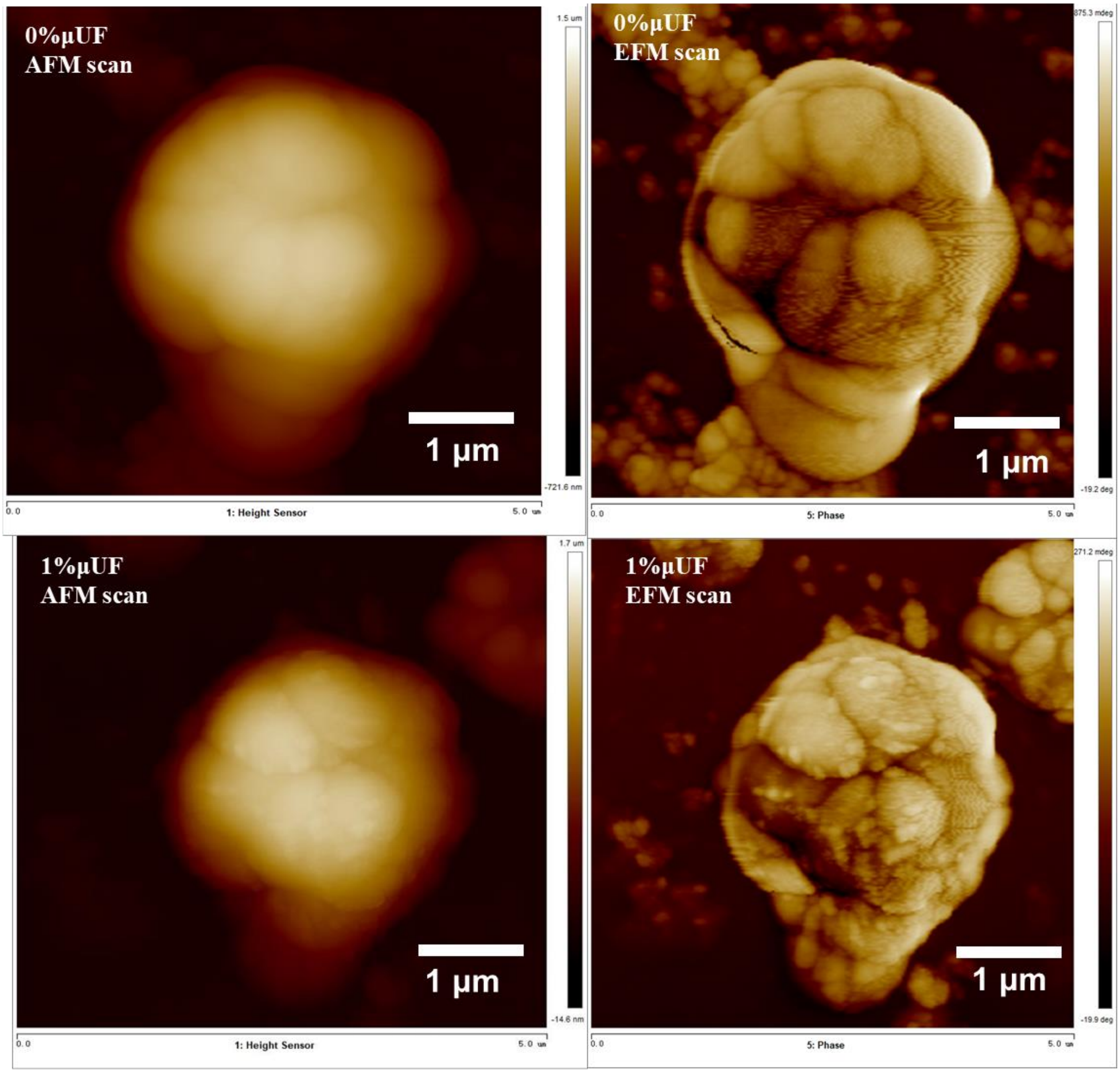

Supplementary Figure 38. 0% µUF and 1% µUF AFM scanning images and EFM scanning 2D images.

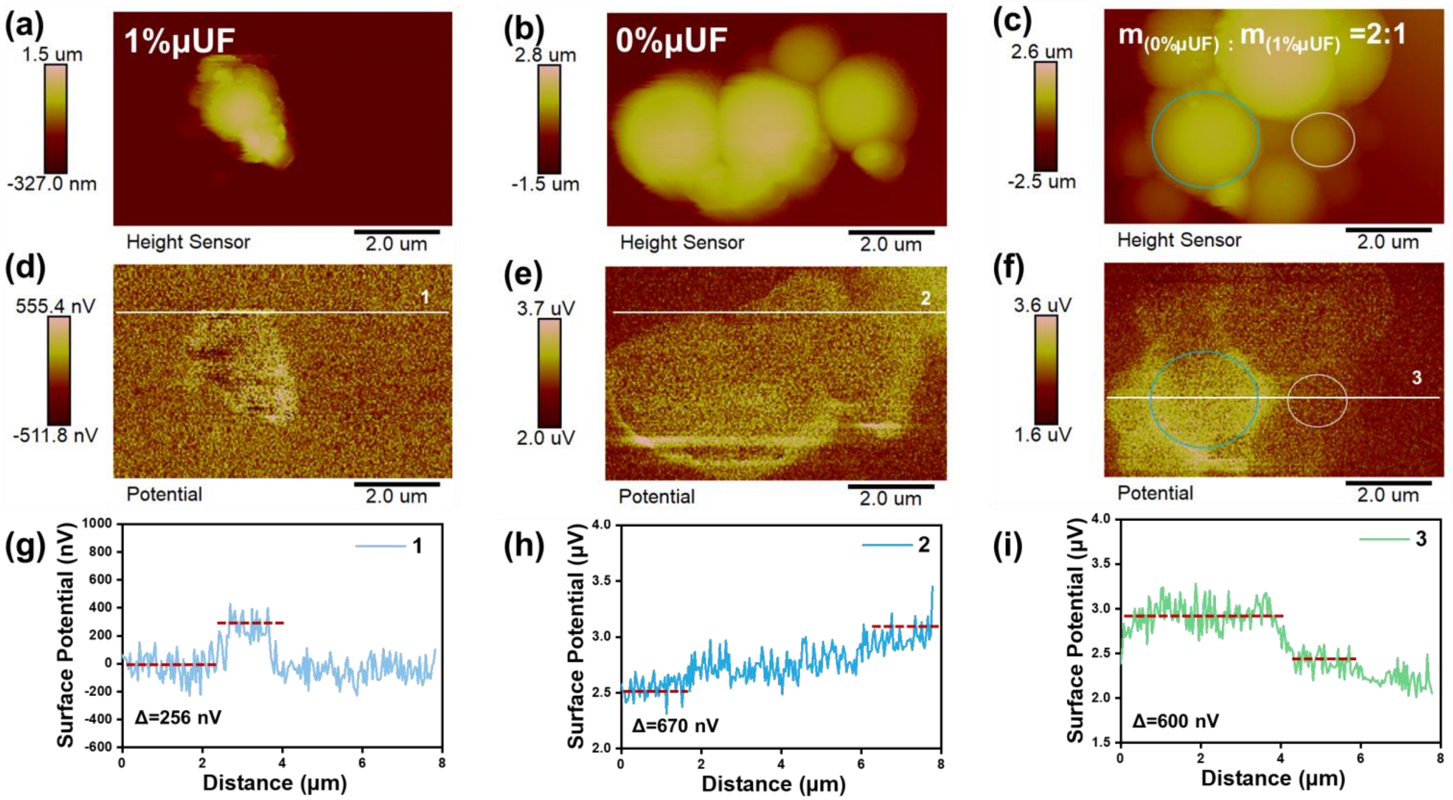

Supplementary Figure 39. Characterization of surface potential variation and charge transfer behavior of 0% μUF and 1% μUF by KPFM. (a-c) AFM topography map, (d-f) corresponding regional surface potential image and (g-i) surface potential profile extracted from surface potential map.

Supplementary References

1. Neese F. Software update: the ORCA program system, version 4.0. *WIREs Computational Molecular Science.* **8**(1)**,** e1327 (2018).

2. Lu T, Chen F. Multiwfn: A multifunctional wavefunction analyzer. *J. Comput. Chem.* **33,** 580-592(2012).
