# Supplementary material for: Urea-formaldehyde resin room temperature phosphorescent material with ultra-long afterglow and adjustable phosphorescence performance
Source: Nat Commun. 2024 May 24;15:4415. doi: 10.1038/s41467-024-48744-w (PMC11126683; doi:10.1038/s41467-024-48744-w)
Supplement: Supplementary file 3 — Description of Additional Supplementary Files [file 41467_2024_48744_MOESM3_ESM.pdf]

Description of Additional Supplementary Files

File Name: Supplementary Data 1
Description: The coordinates of the optimized molecular structure for binding energy calculations

File Name: Supplementary Data 2
Description: The coordinates of the optimized molecular structure for electrostatic potential calculations.

File Name: Supplementary Movie 1
Description: 14DAP/UF, 12DAP/UF and 13DAP/UF showed blue phosphorescence emission under 310nm UV light. After switching off the excitation, bright blue afterglow was observable by the naked eye for several seconds.

File Name: Supplementary Movie 2
Description: Like SV1, 23DAN/UF, 15DAN/UF, 18DAN/UF and 14DAP/UF showed yellow or green phosphorescence emission after switching off the 310nm UV excitation.

File Name: Supplementary Movie 3
Description: 910DAPT/UF showed ultra-long blue afterglow with 47 s under the 310 nm UV light excitation.

File Name: Supplementary Movie 4
Description: Afterglow emission of µUFs under excitation of 310 nm UV light.

File Name: Supplementary Movie 5
Description: Variations in the afterglow of mixtures of 0%µUF and 1%µUF with different masses.

File Name: Supplementary Movie 6
Description: The afterglow of 14DAP/UF, 23DAN/UF and 910DAPT/UF powders after being vulcanized into sheets, respectively. It shows the excellent processing performance of UF-RTPs.
